# Mechano-chemical kinetics of DNA replication: identification of the translocation step of a replicative DNA polymerase

**DOI:** 10.1093/nar/gkv204

**Published:** 2015-03-23

**Authors:** José A. Morin, Francisco J. Cao, José M. Lázaro, J. Ricardo Arias-Gonzalez, José M. Valpuesta, José L. Carrascosa, Margarita Salas, Borja Ibarra

**Affiliations:** 1 Instituto Madrileño de Estudios Avanzados en Nanociencia, IMDEA Nanociencia, 28049 Madrid, Spain; 2 Departamento Física Atómica, Molecular y Nuclear, Universidad Complutense, 28040 Madrid, Spain; 3 Centro de Biología Molecular ‘Severo Ochoa’ (CSIC-UAM), Universidad Autónoma, Cantoblanco, 28049 Madrid, Spain; 4 Instituto Madrileño de Estudios Avanzados en Nanociencia (IMDEA Nanociencia) & CNB-CSIC-IMDEA Nanociencia Associated Unit ‘Unidad de Nanobiotecnología’, 28049 Madrid, Spain; 5 Centro Nacional de Biotecnología (CNB-CSIC), 28049 Madrid, Spain

## Abstract

During DNA replication replicative polymerases move in discrete mechanical steps along the DNA template. To address how the chemical cycle is coupled to mechanical motion of the enzyme, here we use optical tweezers to study the translocation mechanism of individual bacteriophage Phi29 DNA polymerases during processive DNA replication. We determine the main kinetic parameters of the nucleotide incorporation cycle and their dependence on external load and nucleotide (dNTP) concentration. The data is inconsistent with power stroke models for translocation, instead supports a loose-coupling mechanism between chemical catalysis and mechanical translocation during DNA replication. According to this mechanism the DNA polymerase works by alternating between a dNTP/PPi-free state, which diffuses thermally between pre- and post-translocated states, and a dNTP/PPi-bound state where dNTP binding stabilizes the post-translocated state. We show how this thermal ratchet mechanism is used by the polymerase to generate work against large opposing loads (∼50 pN).

## INTRODUCTION

**Figure 1.**
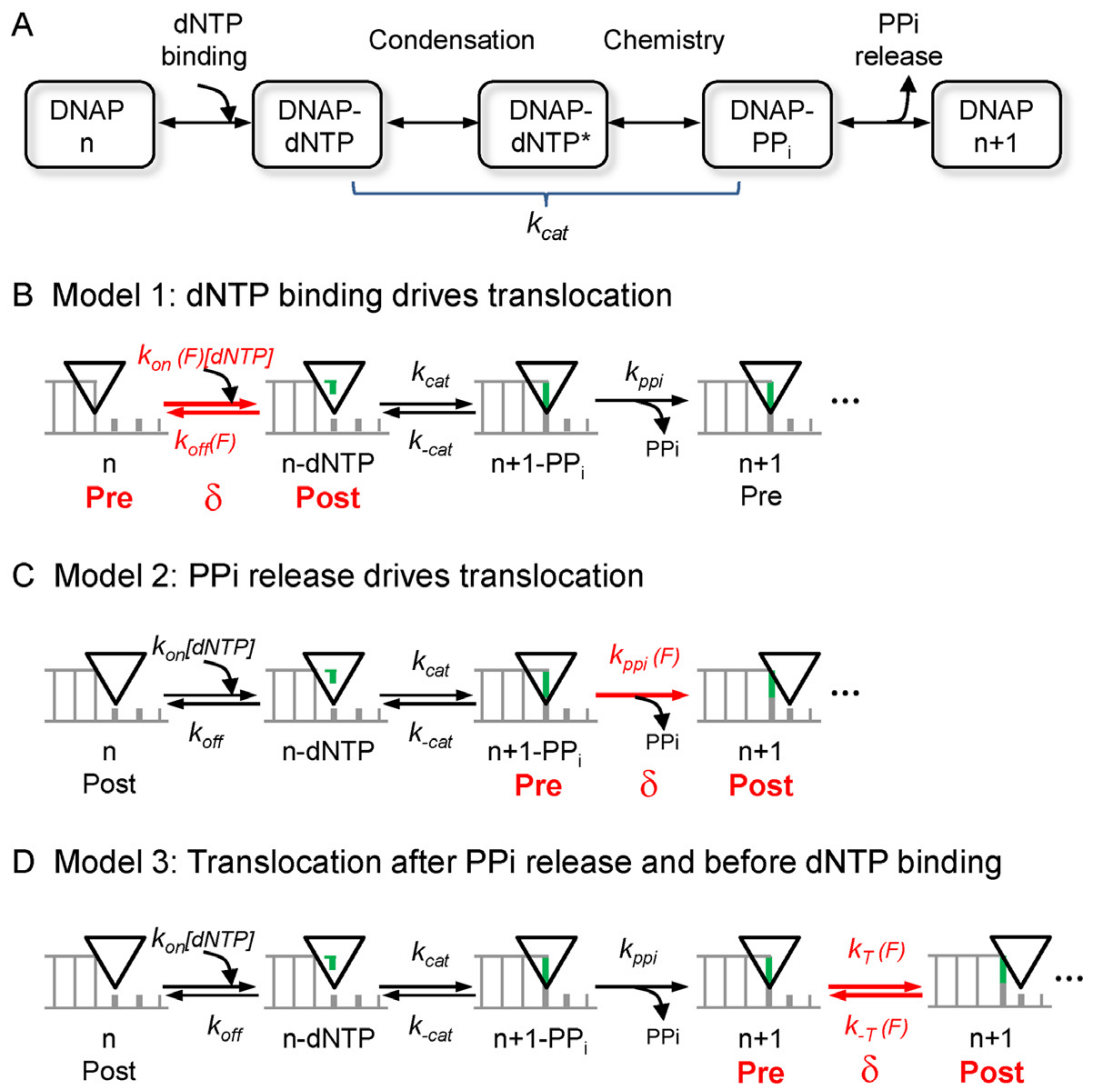
The nucleotide incorporation cycle and alternative kinetic models for DNAP translocation. (**A**) Minimal kinetic mechanism for processive DNA polymerization. After binding the complementary incoming dNTP to the polymerase–DNA complex (DNAPn), rate limiting changes poise the active site ready for catalysis (Condensation). The polymerase then catalyses the incorporation of the dNTP (Chemistry) and pyrophosphate (PPi) is released. At some point in the polymerization cycle, movement to the next template position occurs. For the following models the steps of dNTP condensation and chemistry were grouped within a single rate limiting step (*k*_cat_). (**B**, **C** and **D**) Alternative kinetic models for integrating mechanical translocation within the nucleotide incorporation cycle. Diagrams show the primer-template DNA at the polymerase insertion site (black triangle). Template bases are shown in grey and the incoming nucleotide in green. In each model, the force-dependent translocation occurs at different positions within the cycle: (**B**) Model 1: translocation (δ) is power-stroked by dNTP binding. (**C**) Model 2: translocation (δ) is power-stroked by PPi release. (**D**) Model 3: a Brownian ratchet mechanism where reversible fluctuations between pre- and post- translocated DNAP states (δ) occur after PPi release and before dNTP binding. Rate constants are defined in the main text and Supplementary Data.

Replicative DNA polymerases (DNAPs) work as molecular machines that catalyze template-directed DNA replication in a processive manner. These enzymes share a common metal-ion catalytic mechanism for the incorporation of the complementary deoxy-nucleoside triphosphate (nucleotide or dNTP) to the 3′ end of the growing primer strand (1,2). They also present similar structural organization of the polymerization domain, with three subdomains referred to as the thumb, fingers and palm (3). The processive nucleotide incorporation cycle involves a series of conformational changes and intermediate states that couple the chemical steps of the reaction to the mechanical translocation of the polymerase relative to the DNA by one nucleotide at a time (4). In each reaction cycle, the most evident conformational change is the rotation of the fingers subdomain between open and closed conformations. Following the initial binding of the polymerase to the DNA, a minimal model of nucleotide incorporation starts with the binding of an incoming dNTP to form a ternary dNTP–polymerase–DNA complex (Figure 1A). Binding of the complementary dNTP stabilizes the closed conformation of the fingers through a series of intermediate states relevant for nucleotide selection (5–9). Then rate-limiting non-covalent transformations activate the ternary complex to form an active site poised for catalysis (5,10–20). This is immediately followed by a rapid phosphoryl transfer reaction: the new phosphodiester bond is formed with concomitant pyrophosphate (PPi) cleavage from the nucleotide. The cycle is completed by the PPi release and motion of the fingers from the closed to the open state (21,22). During this cycle the polymerase should translocate from the ‘pre-translocated’ state, where the active site is occupied by the newly added nucleotide, to the next 3′-OH primer terminus or ‘post-translocated’ state. The translocation step facilitates the processive movement of the polymerase along the template DNA and it is crucial to maintain genetic integrity. An accurate, highly coordinated stepping is necessary to prevent frame-shift mutations and to modulate the balance toward the ‘editing’ or exonuclease mode in which mismatched bases are excised by the polymerase (16,21,23).

Despite the wealth of kinetic and structural information available on the catalytic cycle of DNAPs, little is known about the kinetics and energetics of the fundamental step of translocation and its integration in the nucleotide incorporation cycle. Based on extensive biochemical, structural and single molecule studies on RNA polymerases two classes of general models have been proposed to explain the coupling between chemical and mechanical steps during the activity of nucleic acid polymerases. The first class argues for a tight-coupling mechanism between the chemical and mechanical steps of the reaction where either binding of the incoming nucleotide (Figure 1B) or release of the PPi product (Figure 1C) promote translocation (4,24–28). The second class proposes a loose-coupling mechanism between chemical catalysis and mechanical translocation, in which the thermal diffusion of the polymerase along the DNA between the pre- and post-translocated states is directionally rectified by nucleotide binding (Figure 1D) (29–35). Recent single molecule experiments with binary DNAP–DNA complexes transiently bound to a nanopore suggested that the latter mechanism could also apply to DNAPs (36–38). To specifically determine whether any of these general mechanisms explain also the mechano-chemistry of DNAP, we used optical tweezers to measure the combined effect of nucleotide concentration and load aiding and opposing forward motion, on the real-time kinetics of individual Phi29 DNAP molecules replicating the DNA template in a processive manner.

The replicative DNA polymerase from the bacteriophage Phi29 presents great processivity, a strong strand displacement activity and an associated 3′–5′ exonuclease activity, which make this protein an excellent model system for leading strand DNA synthesis catalyzed in more complex replisomes (39,40). Recent single molecule manipulation studies showed that mechanical tension applied to the DNA modulates the proofreading and strand displacement activities of this protein (41–43). In the current work, we applied mechanical force or load directly to the polymerase. In this case, load is expected to specifically interfere with the mechanical step of the protein translocation along the DNA (44). Studying the combined effect of load and dNTP concentration on the reaction kinetics allowed us to provide a detailed pictured of the coupling between the chemical and mechanical steps of the replication reaction.

Our data favors a loose-coupling mechanism between chemical catalysis and mechanical translocation where a dNTP/PPi-free polymerase–DNA complex can thermally diffuse between the pre- and post-translocated states. Binding of the incoming nucleotide is critical to stabilize the post-translocated dNTP-bound conformation. In this conformation, the polymerase can generate force and work against surprisingly large opposing load (∼50 pN). In addition, we calculated the rates and force dependencies of the main steps of the nucleotide incorporation cycle. Interestingly, the rate measured for the forward translocation step is only ∼5 times faster than the rate-limiting step, indicating that the polymerase spends a considerable amount of time at the dNTP/PPi-free conformation. This observation suggests a possible role of the translocation step in modulating the balance toward the ‘editing’ or exonuclease reaction.

## MATERIALS AND METHODS

### Optical tweezers experiments

Stalled, binary Phi29 DNAP-DNA replication complexes were formed in bulk by incubation (5 min at 22°C) of the biotin tagged polymerase (0.3 ng) with a gapped DNA substrate labeled with digoxigenin (3 ng) in a reaction buffer containing: 50 mM Tris–HCl (pH 7.5), 20 mM ammonium sulphate, 7 mM 2-mercaptoethanol, 4% glycerol (w/v), 0.025% Tween20 (w/v), 0.1 mg/ml bovine serum albumin (BSA). The stalled complexes were immobilized between streptavidin and anti-digoxigenin-coated beads (Spherotech) in the optical tweezers and the reaction was started by flowing the dNTPs (2, 5, 10, 50, 100, 200 and 500 μM) diluted in the reaction buffer supplemented with 10 mM MgCl_2_ and 1 μM or 1 mM of PPi. A detailed description of protein labelling, *in vitro* replication assays and template preparation is given in Supplementary Methods and Supplementary Figures S1 and S2.

Data were collected in a dual-beam optical tweezers at 60 Hz at 22 ± 1°C (45). Measurements were carried out in two modes: ‘constant force feedback’, in which the distance between the beads was adjusted to maintain a constant load in the polymerase-DNA, and ‘no feedback’, in which the distance between the optical trap and the pipette was held constant. A total of 305 independent replication activities were recorded and analyzed.

To study the process of product (PPi) release we measured replication activities at 2 μM dNTP with 1 μM or 1mM PPi concentrations. These activities were collected in a dual trap optical tweezers with an enhanced position resolution at 1 kHz and 28 ± 0.5°C in the ‘no feed-back’ operation mode, Supplementary Figure S3 (46).

### Data analysis

The number of nucleotides incorporated as a function of time at a particular opposing load was obtained by dividing the observed distance decrease between the beads by the average distance between single- (for primer extension conditions) or double-stranded (for strand displacement conditions) nucleotides at that load (Supplementary Methods and Supplementary Figure S4). At a constant aiding load, the replication activity increases the length of the downstream DNA, both during primer extension and strand-displacement conditions. In this case, for both replication conditions, the number of nucleotides incorporated as a function of time was obtained by dividing the distance change between the beads by the average distance between double-stranded nucleotides at a particular load (Supplementary Methods). The expected change in distance corresponding to primer extension replication at a particular constant load was calculated by multiplying the length of the ssDNA template (229 nt) by the average distance between single- (opposing load conditions) or double-stranded (aiding load conditions) nucleotides at that load.

The average rates (with and without pauses) were determined by a line fit to the traces showing the number of replicated nucleotides versus time. The final rate at each load was obtained by averaging over all of the traces at similar load values (± 2.5 pN). Instantaneous replication rates were obtained from a linear fit of the number of replicated nucleotides over a sliding time window of 0.7 s (50 data points) for all velocities. Velocity distributions were determined from the histogram of the instantaneous replication rate using a bin of five nucleotides per second. Pause events were identified with a resolution of 0.4–0.8 s following the method described elsewhere, Supplementary Figure S5 (42,43).

## RESULTS

### Experimental design and detection of single DNA replication activities

**Figure 2.**
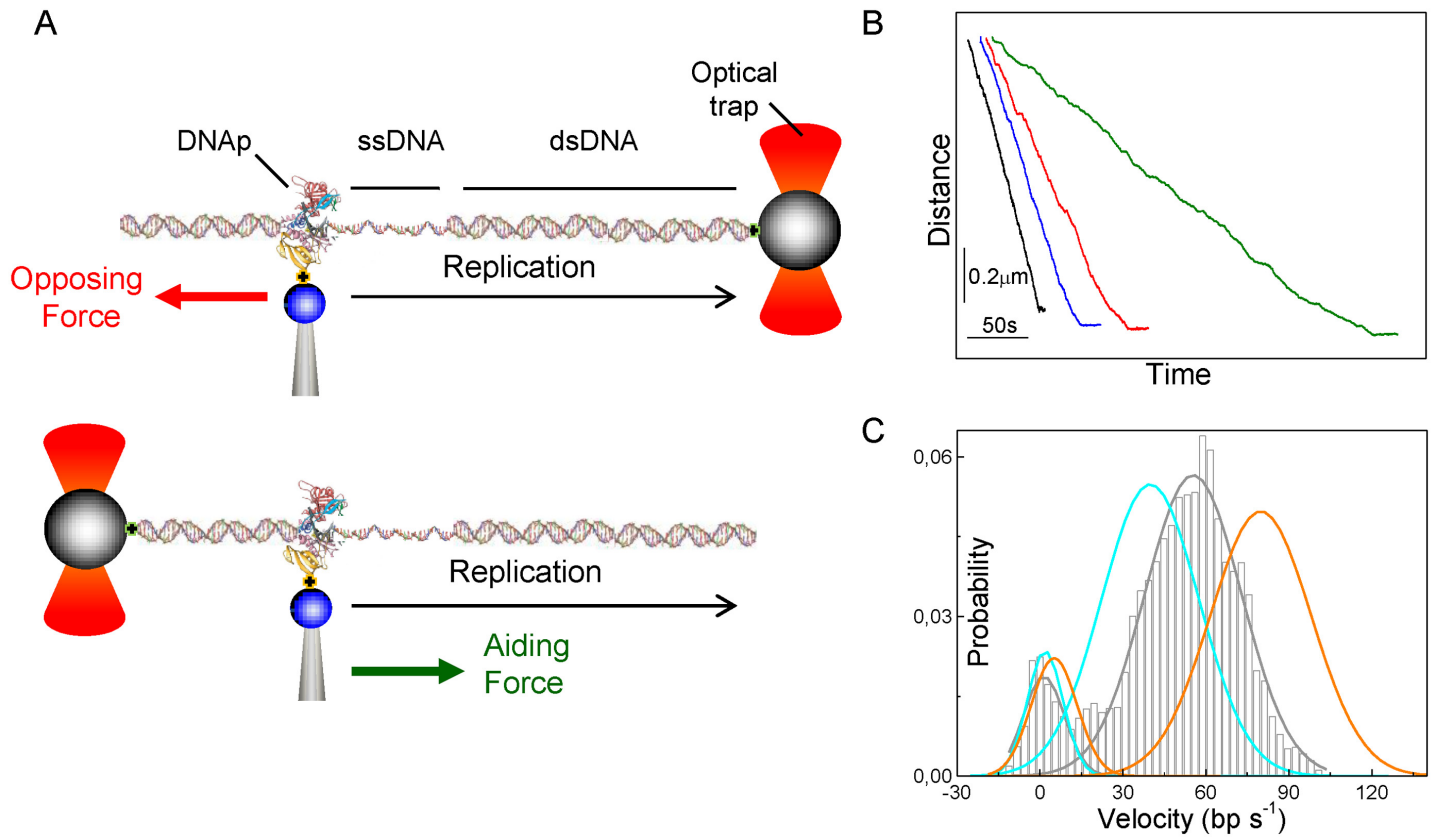
Experimental configuration and replication records. (**A**) Cartoons of experimental configurations for application of load opposing (top) or aiding (bottom) translocation (not to scale). A single polymerase molecule is attached to a bead (blue) held on a micropipette and tethered via the upstream (opposing force) or the downstream (aiding force) DNA template to a bead (gray) in the optical trap. At constant opposing force (red arrow) the length of the DNA tether shortens, while a constant aiding force (green arrow) the length of the DNA tether increases, as replication proceeds. Supplementary Figure S1 shows schematic diagrams of the DNA constructs. (**B**) Representative, independent replication traces measured at constant opposing forces of −3, −10, −17 and − 26 pN (black, blue, red and green) showing the DNA tether length versus time (50 μM dNTP). The traces include both the initial primer extension and the following strand displacement replication modes. The expected distance change for each replication mode is described in the ‘Materials and Methods’ section. The traces are offset for clarity. (**C**) Distribution of instantaneous velocities during strand displacement replication conditions recorded at 500 μM dNTP and opposing loads of −10 (orange, *N* = 8), −15 (gray, *N* = 8) and −25 pN (light blue, *N* = 7). *N* represents the number of independent traces used to generate the histogram. Means and standard error of the mean (SEM) in bp/s are for the ‘positive velocity peak’ 79 ± 6 (−10 pN), 55 ± 6 (−15 pN) and 39 ± 6 (−25 pN), and for the ‘zero velocity peak’ ∼4 ± 3 for all three distributions. The ‘positive velocity peak’ corresponds to the active state of the polymerase, whereas the ‘zero velocity peak’ corresponds to the inactive, paused state. The original histogram is shown for −15 pN conditions. See Supplementary Methods and Figures S5 and S6 for pause identification procedures.

Replication by single DNAP molecules was recorded using a dual-beam optical tweezers (45). A binary Phi29 DNAP–DNA replication complex containing a biotin tag on the amino terminus of the polymerase was attached to a streptavidin-covered bead on top of a micropipette. The upstream or downstream end of the template DNA was attached to the bead in the laser trap, allowing the application to the replicating enzyme of hindering or assisting load, respectively (see ‘Materials and Methods’ section and Figure 2A). Replication activities were measured along the full length of DNA molecules containing downstream the 3′ end polymerase binding site a single-stranded stretch of 229 nucleotides followed by 3487 base pairs of double-stranded DNA (Figure 2A and Supplementary Figure S1). This template design, together with the extraordinary processivity and potent strand displacement activity of the Phi29 DNA polymerase, allowed measuring replication during primer extension and strand displacement conditions. During strand displacement replication conditions, the polymerase replicates the template and at the same time displaces the complementary strand. The experiments began by holding a constant force on the replication complex, then activity was started by introducing the four dNTPs/Mg^2+^ into the fluid chamber and determined from the decrease (hindering loads) or increase (aiding loads) in the contour length of the DNA tether (Figure 2B). These length changes were subsequently converted to the number of nucleotides incorporated as a function of time at a given force (see ‘Material and Methods’ section). In addition, we checked that the biotin-tagged polymerase retains *in vitro* the polymerization activity and polymerization/exonucleolysis balance characteristic of the untagged wild-type polymerase (Supplementary Figure S2).

### Force and dNTP/PPi dependence of the nucleotide incorporation cycle

**Figure 3.**
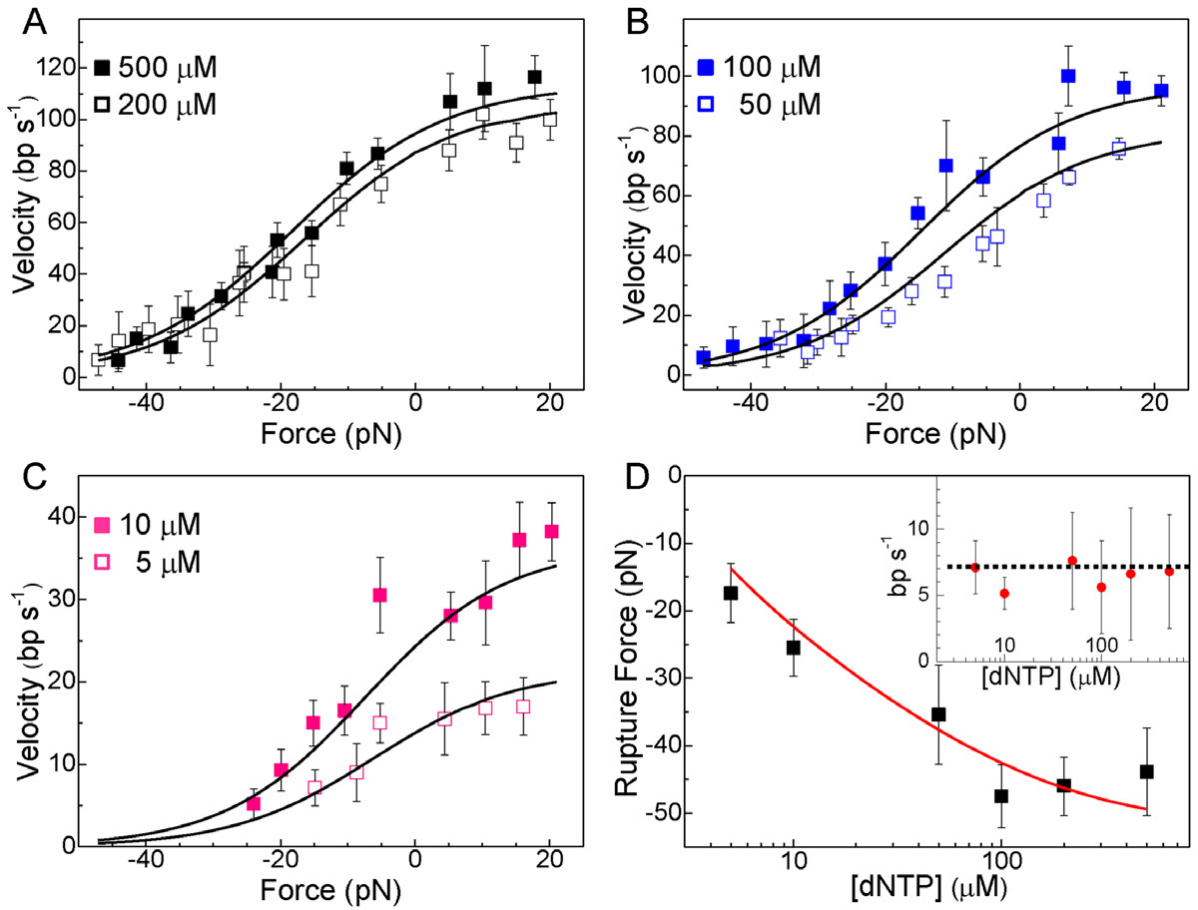
Force–velocity measurements and rupture force. (**A**, **B** and **C**) Average replication rates (without pauses) as a function of force measured at 500, 200, 100, 50, 10 and 5 μM dNTP concentrations. Data correspond to strand displacement replication conditions. No significant differences were found between the force–velocity relationships corresponding to the primer extension and strand displacement replication conditions (Supplementary Figure S7). Negative forces correspond to hindering loads; positive forces to assisting loads. Solid black lines represent the fits of the data with the Michaelis–Menten expression for velocity (Equation 1) including Equations (3) and (4). The results of the fits yielded the values of the coefficients *a, b, r, s, d*_b_ and *d*_s_ shown in Table 1. (**D**) Rupture force as a function of dNTP concentration. Solid red line represents the prediction of the data from the fits of the force-velocity relationships. The inset shows the average velocity measured before polymerase–DNA detachments as a function of dNTP concentration. The dotted black line shows the squared error weighted average rate, 7 nt/s. For all figures error bars correspond to the SEM at each force.

Replication velocities were measured over a wide range of assisting and hindering loads (20 to −50 pN, respectively) at seven dNTPs concentrations ranging from 2 to 500 μM with inorganic pyrophosphate (PPi) fixed at 1 μM (unless otherwise noted). At saturated dNTP concentrations, the velocity at the lowest loads was in excellent agreement with the replication rates reported previously for this polymerase (*v* ∼80 nt/s) (42–43,47–49). The force–velocity plots showed that for all dNTP concentrations the average replication velocity without pauses, *v*, is slightly favored by assisting load and decreased gradually towards stalling as hindering load increased (Figure 3A–C). We did not find significant differences between the force–velocity relationships corresponding to the primer extension and strand displacement replication conditions, suggesting that the mechanism used by the polymerase to separate the two strands of the DNA is not affected by load (Supplementary Figure S7). Pauses or velocity changes associated with the transition between the two replication modes were also not found at any force (Figure 2B). A detailed analysis of pause behavior revealed that off-pathway pauses are independent of load (and therefore have no associated motion) and do not affect the overall force–velocity relationships (Supplementary Figures S5 and S6).

In order to determine the magnitude of the opposing forces required to stall the polymerase advance at different dNTP concentrations, we also recorded replication activities in the ‘no-feedback mode’ of operation. In this mode, force opposing the polymerase movement increases gradually as replication proceeds along the template (Supplementary Figure S8A). However, we could not determine the mechanical stalling of the reaction since the attachments between the beads were abruptly disrupted before the actual stalling of the protein was reached (Supplementary Figure S8A). For all dNTP concentrations detachment occurred during active replication with an average rate of ∼7 nt/s (Figure 3D inset). Interestingly, the load required for detachment depends on the dNTP concentration (Figure 3D), strongly suggesting that detachment events correspond to the disruption of the polymerase–DNA interactions (since protein–bead and DNA–bead connections should be independent of dNTP concentration). At saturating dNTP concentrations, the Phi29 DNAP replicates the template against large hindering loads of ∼50 pN (Figure 3A), pointing out the extraordinary stability of the replication complex against opposing load. In the aiding geometry, however, detachment of the polymerase–DNA interactions occurred, for all dNTP concentrations, at loads right above 20 pN (Supplementary Figures S8B and S8C). These results suggest an asymmetry in the strength of the polymerase–DNA interactions along the experimental pulling geometry. We note that, for all experimental conditions tested, we did not observe reverse movements or ‘negative velocities’ due to a processive exonuclease activity of the polymerase.

**Figure 4.**
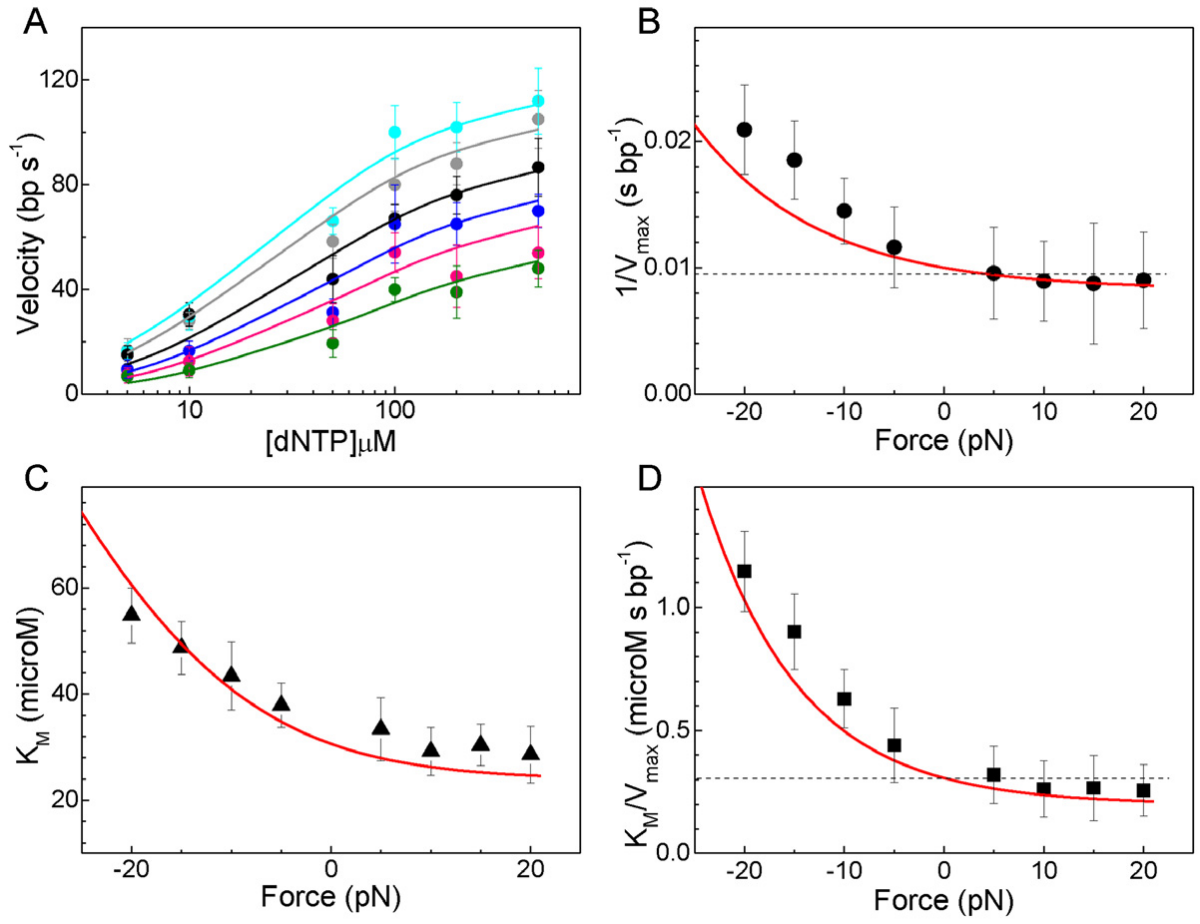
Force dependence of the Michaelis–Menten parameters. (**A**) Average replication rates (without pauses), during strand displacement replication conditions, as a function of dNTP concentration measured at several aiding and opposing loads: 20, 5, −5, −10, −15 and −20 pN (light blue, gray, black, dark blue, pink, and green). Error bars correspond to the SEM. Nonlinear least-square fits to the Michaelis–Menten equation (Equation 1) are shown (solid lines). (**B**) Inverse of *V*_max_ versus load as determined from the fits of the data in (A). (**C**) *K*_M_ as determined from the fits of the data in (A). (**D**) The ratio *K*_M_/*V*_max_ (or 1/*k*_b_) plotted against force. For (B), (C) and (D): error bars were obtained from the fits. Solid red lines show predictions from the best fit of the force–velocity relationships using the Brownian ratchet model (Model 3). Power-stroke models, where dNTP binding (Model 1) or PPi release (Model 2) drive translocation, predict load independent 1/*V*_max_ or *K*_M_/*V*_max_, respectively (dashed black lines in panels B and D).

Fitting the velocity dependence on dNTP concentration, [dNTP], at different loads to the Michaelis-Menten equation (Figure 4A)

(1) \begin{equation*} v = V_{{\rm max}} \left (^{[{\rm dNTP}]}/ _{K_M + [{\rm dNTP}]} \right ) \end{equation*}

(at 1 μM PPi concentration the contribution of the reverse reaction is insignificant, see below), revealed the values for the velocity at saturated dNTP concentrations, *V*_max_(0) = 112 ± 11 nt/s, the apparent nucleotide binding constant of the reaction*, K*_M_(0) = 31 ± 3 μM and their respective load dependencies; while *V*_max_ is reduced (or 1/*V*_max_ increased), *K*_M_ is raised by hindering loads (Figures 4B and 4C, respectively). Therefore, considering a load independent coupling efficiency of one dNTP hydrolysis per step (50,51), the effective rate of dNTP binding, defined as *k*_b_(*F*) *= V*_max_(*F*)/*K*_M_(*F*), decreases with load (or 1/*k*_b_ increases with load, Figure 4D). Thus, the application of hindering load lowers both the maximal translocation rate and the effective rate of dNTP binding of the polymerase. Note that the inverses of *V*_max_ and *k*_b_ provide expressions with simpler relations among the characteristic times (inverse of the rates) and the processes involved during the cycle (Supplementary Data). These simple relations provide a straightforward interpretation of the observations and their implications (see kinetic expressions below).

Finally, to study the process of product release we measured the effect of PPi on replication velocity at a sub-saturating dNTP concentration of 2 μM. Increasing the PPi concentration 1000-fold (to 1 mM) had no significant effect on the average replication velocity measured at different loads (Supplementary Figure S3). In agreement with bulk studies, these results indicate that under our experimental conditions the equilibrium constant for PPi release is very large (52,53). Accordingly, this step was considered largely irreversible for all the mechano-chemical models contemplated in this work (Figure 1B–D).

### Coupling between mechanical and chemical cycles

To determine the load-dependent step, or the step of the nucleotide incorporation reaction related to movement, we considered a minimal nucleotide incorporation cycle where the rate-limiting activation of the ternary complex and the following rapid chemical steps were grouped within a single rate limiting step (*k*_cat_, Figure 1A). According to biochemical and structural studies no significant conformational changes within the polymerase–DNA complex occur during these steps (2,4,21,24,52,54), indicating that translocation is not expected to occur concomitantly to these reactions. Hence, in this minimal nucleotide incorporation cycle (Figure 1A), three different general models might explain the coupling mechanism between the chemical and mechanical steps of the reaction. In Model 1 (Figure 1B), translocation is power-stroked by dNTP binding, in Model 2 (Figure 1C), translocation is power-stroked by PPi release and in Model 3 (Figure 1D), translocation occurs by thermal diffusion of the polymerase–DNA complex after PPi release and before dNTP binding. Note that we have assumed that a single step is associated with translocation (in other words, the cycle present a single force dependent step). We considered that the forward and reverse rates of the translocation step present an Arrhenius-like dependence on force, $k_i \left( F \right) = k_i \left( 0 \right)e^{\frac{{F \cdot d_i }}{{k_{\rm B} T}}}$, where *F* is the applied load, *k*_B_ is Boltzmann's constant, *T* is the temperature, and *d_i_* is the effective distance over which the applied load acts on translocation (44). Please, refer to Supplementary Information for additional alternative models.

#### Data is not compatible with power-strokes models (Model 1 and Model 2)

The observed force dependency of 1/*V*_max_ rules out Model 1, in which translocation is driven by the nucleotide binding reaction (Figure 1B). In this model, load opposing translocation would only affect the nucleotide binding and/or unbinding rates, *k*_on_(*F*) [dNTP] and *k*_off_(*F*) respectively. A direct consequence of this model is that the maximum replication velocity at saturated dNTP concentrations, *V*_max_, should not depend on force. This is because, by definition, *V*_max_ does not depend on the nucleotide binding and/or unbinding rates (44,55–57), which are the only force dependent rates of the cycle in Model 1 (Supplementary Data). The observed force dependency of 1/*V*_max_ argues directly against this conclusion (Figure 4B) and therefore, a power stroke mechanism driven by the nucleotide binding reaction can be directly excluded from the data.

The observed force dependency of 1/*k*_b_(*F*) rules out a mechanism where translocation occurs during PPi release, Model 2 (Figure 1C). In this model, the rate of PPi release *k*_ppi_(*F*), is the only force dependent rate of the nucleotide incorporation cycle. According to this model the inverse of *k*_b_(*F*) (or *K*_M_ (*F*)/*V*_max_ (*F*)) can be written as (Supplementary Data)

(2) \begin{equation*} \frac{1}{{k_{\rm b} (F)}} = \frac{1}{{k_{{\rm on}} }}\left[ {1 + \frac{{k_{{\rm off}} }}{{k_{{\rm cat}} }}\left( {1 + \frac{{k_{ - {\rm cat}} }}{{k_{{\rm ppi}} \left( F \right)}}} \right)} \right]\end{equation*}

Kinetic studies of Family A and B DNAPs showed that *k*_ppi_ is typically ∼10^3^ times faster than the reverse of the rate limiting step of the reaction, *k*_*−*cat_ (14,16,21,58–61). This implies that the ratio *k*_*−*cat_/*k*_ppi_(*F*) is <<1 and therefore, for a mechanism where translocation is coupled to PPi release, 1/*k*_b_(*F*) should be largely independent on force (considering *d_i_* ∼0.34 nm). This prediction contrasts with our data showing a clear load dependency of 1/*k*_b_(*F*) (Figure 4D), arguing against a direct connection between the PPi release step and mechanical translocation.

#### Data is compatible with a Brownian-ratchet model (Model 3)

The alternative Model 3 considers that translocation occurs by thermal diffusion in an additional step located after PPi release and before dNTP binding (Figure 1D). In this case, the force dependent rates of the reaction are the forward and backward translocation rates, *k*_T_(*F*) and *k*_*−*T_(*F*), respectively. In contrast to the other two models described above, the kinetic expressions for the Michaelis-Menten parameters *V*_max_, *K*_M_ and their ratio, *k*_b_, derived from Model 3 allow initially a force dependent behavior for both 1/*V*_max_(*F*) and 1/*k*_b_(*F*) (Supplementary Data). In order to test the validity of this model to explain the data, we first grouped in the 1/*V*_max_(*F*) and 1/*k*_b_(*F*) expressions the force independent and force dependent terms in the following way:

(3) \begin{equation*} \frac{1}{{V_{{\rm max}} (F)}} = a + b \cdot e^{F \cdot d_{\rm b} /(k_{\rm B} T)} ,\end{equation*}

(4) \begin{equation*} \frac{1}{{k_{\rm b} (F)}} = \frac{{K_{\rm M} (F)}}{{V_{{\rm max}} (F)}} = r + s \cdot e^{F \cdot d_{\rm s} /(k_{\rm B} T)} ,\end{equation*}

where the coefficients *a, b, r, s*, are given in terms of the rates of the cycle and, the coefficients *d*_b_ and *d*_s_ define the force dependency or effective distance over which force acts on translocation (see below and Supplementary Data).

Then, we included Equations (3) and (4) in the Michaelis-Menten velocity definition (Equation 1) to obtain *v*(*F*, [dNTP]) and used it to fit simultaneously the force-velocity relationships for all dNTP concentrations (Figure 3A–C). The results of the fits yielded the values of the coefficients *a, b, r, s, d*_b_ and *d*_s_ (Table 1). Importantly, these coefficients predicted very well the observed force dependency for 1/*V*_max_(*F*), *K*_M_(*F*) and 1/*k*_b_(*F*) (Figures 4B, 4C and 4D, respectively) and the observed dNTP concentration dependence of the detachment load, red solid line in Figure 3D (Supplementary Data).

**Table 1.** Left: Best fit values of the six coefficients of the force–velocity relationships given by the Michaelis–Menten expression for velocity including Equations (3) and (4)

*a* (s)	0.0084	*k*_on_ (s^−1^ μM^−1^)	5
*b* (s)	0.0015	*k*_cat_ (s^−1^)	120
*r* (s)	0.19	*k*_T_ (0) (s^−1^)	670
*s* (s)	0.12	k_−T_ (0) (s^−1^)	420
*d*_b_ (nm)	0.35	*K*_δ_ (0) = *k*_T_(0)/*k*_-T_(0)	1.59
*d*_s_ (nm)	0.40	*d*_T_ (nm)	0.35
		*d*_−T_ (nm)	0.05
		*δ* (nm)	0.40

Right: Kinetic rates, equilibrium constants and force dependencies derived for the nucleotide incorporation cycle described by the Brownian ratchet model (Model 3).

The coefficients *a, b, r*, and *s* are directly related to the rates of several steps of the kinetic cycle (Supplementary Data)

\begin{equation*} a \sim \frac{1}{{k_{{\rm cat}} }};\;b = \frac{1}{{k_{{\rm T}} (0)}};\;r \sim \frac{1}{{k_{{\rm on}} }}\;{\rm and}\;\frac{s}{r} = \frac{{k_{ - {\rm T}} \left( 0 \right)}}{{k_{\rm T} \left( 0 \right)}}.\end{equation*}

Whereas, the coefficients *d*_b_ and *d*_s_ are directly related with the characteristic distances from the pre-translocation position to the transition state (*d*_T_), and from the transition state to the post-translocation state (*d*_*−*T_) as follows (Supplementary Data):

\begin{equation*} d_{\rm b} = d_{\rm T} \;{\rm and}\;d_{\rm s} = d_{\rm T} + d_{ - {\rm T}} .\end{equation*}

These relationships allowed us to obtain directly the values of several of the main rates and force dependencies of the nucleotide incorporation cycle which in turn, are fully consistent with the Brownian ratchet mechanism for translocation proposed by Model 3.

Regarding the translocation step, the above relationships imply that *k*_T_(0) = 670 s^−1^, *k*_−T_(0) = 420 s^−1^, *d*_T_ *=* 0.35 nm, and *d*_*−*T_ = 0.05 nm (Table 1), where *k*_T_(0) and *k*_*−*T_(0) correspond to the load dependent forward and backward translocation rates in the absence of load, respectively, and *d*_T_ and *d*_*−*T_ to their respective associated characteristic distances. Two important conclusions about the translocation step can be obtained from these values: (i) the effective step size affected by load *d*_s_ = *d*_T_ + *d*_−T_ = 0.40 nm, is compatible with the expected distance between the pre- and post-translocated states, *δ* ∼ 0.34 nm (34,54,62), and therefore, *d*_s_ is equivalent to *δ*. We note that the fingers conformational change that may accompany the translocation step is probably perpendicular to the direction of translocation and its magnitude cannot be directly measured with our experimental approach; 2) the equilibrium constant of the translocation step *K*_δ_ (0) = *k*_T_(0)/*k*_-T_(0) = 1.59, implies a small associated average free energy change for translocation, Δ*G*_trans_ = −ln*K*_δ_*k*_B_*T* = −0.46*k*_B_*T*, supporting thermal diffusion as a plausible mechanism to explain the relative movement between the polymerase and the DNA.

In addition, we obtained the apparent rate of dNTP binding, *k*_on_, from the relationship *k*_on_ ∼1/*r =* 5 μM^−1^ s^−1^ (Table 1). This value is consistent with the dGTP binding rate (*k*_on_ ∼17 μM^−1^ s^−1^) recently determined for the Phi29 DNAP (36). Furthermore, we obtained the rate of the nucleotide condensation and catalysis step, *k*_cat_, from the relationship *k*_cat_ ∼1/*a* = 120 s^−1^ (Table 1). We note that since the main contribution to $1/V_{{\rm max}}$ at zero force comes from the value of the parameter *a* (*a* > *b*, Table 1), *k*_cat_ corresponds to the rate limiting step of the reaction at saturated dNTP concentrations. Importantly, this result meets our initial condition that considered, based on kinetic and structural studies on several DNAPs (5,10–20), the steps comprising *k*_cat_ as the rate limiting step of the cycle. The only forward rate we could not derive from the fits is the rate of PPi release, *k*_ppi_, which has been determined for many DNAPs as one of the fastest rates of the nucleotide incorporation cycle, *k*_ppi_ = 10^3^–10^4^ s^−1^ (14,16,21,58–61).

Altogether, the results from the fits are consistent with the kinetic model for nucleotide incorporation proposed by Model 3, where translocation occurs by thermal diffusion of the polymerase–DNA complex between pre- and post-translocated states separated by a distance equivalent to the mean rise per base found in B-DNA.

## DISCUSSION

Our single-molecule, real-time observations of actively translocating Phi29 DNAP molecules under varying loads and dNTP concentrations reveal the mechanism of coupling between chemical and mechanical energy. Our data eliminates tight-coupling mechanisms between chemical catalysis and mechanical translocation, where conformational changes during dNTP binding or PPi release are used directly to power translocation during DNA replication (Models 1 and 2, Figure 1B and C). Whereas these models predict force-independent 1/*V*_max_ or 1/*k*_b_ respectively, the data reveal that both of these parameters are dependent on force (Figures 4B and 4D).

**Figure 5.**
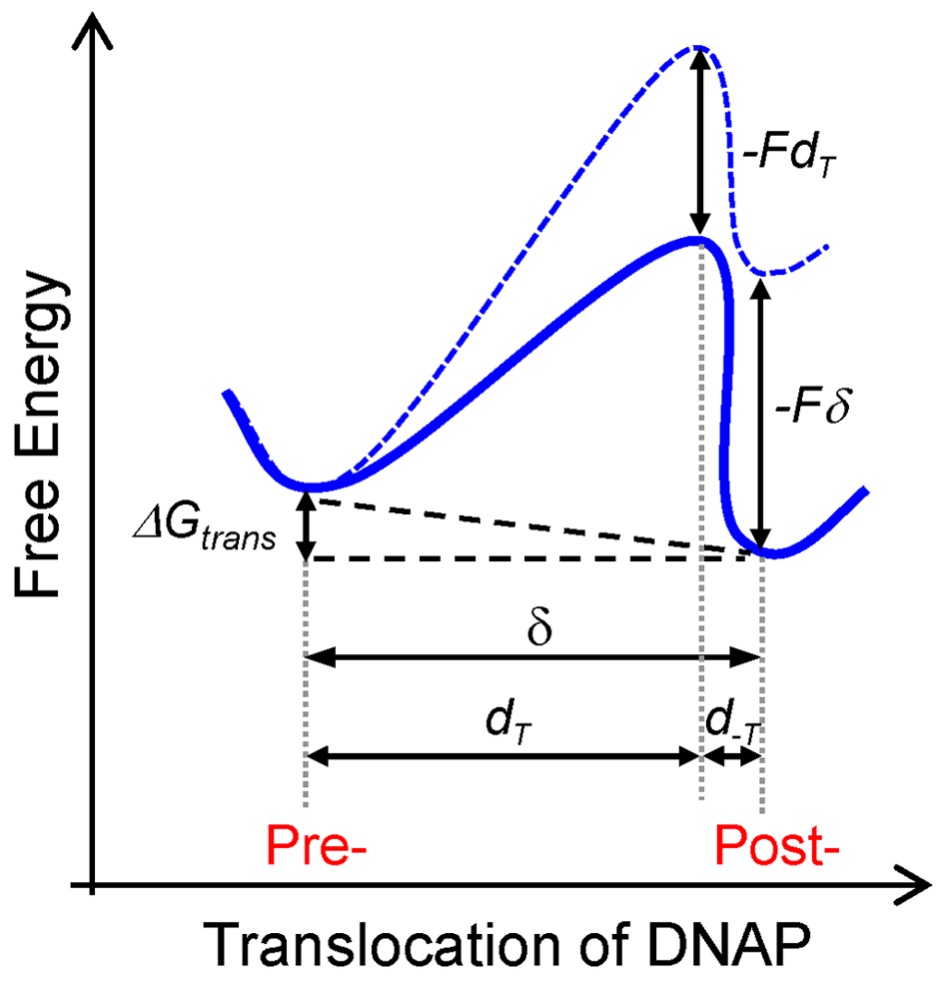
Effect of opposing load on the free energy landscape for translocation. According to the Brownian ratchet model (Model 3), in the absence of load the post-translocated state is favored by Δ*G*_trans_ ∼ −0.46 *k*_B_*T* (solid blue line). Opposing load tilts the energy landscape (dotted blue line) by an amount equal to the work performed against the applied load, −*Fδ*, where *δ* corresponds to the equilibrium distance between the pre- and post-translocated states (*δ* = 0.4 nm) and the transition state barrier is raised by an amount –*Fd*_T_. Application of load opposing translocation shifts the equilibrium towards the pre-translocated state. *d*_T_ is the characteristic distance from the pre-translocation position to the transition state and *d*_*−*T_ is the distance from the transition state to the post-translocation state (Table 1).

Instead, our data is compatible with a loose-coupling model between chemical catalysis and mechanical translocation in which translocation occurs following the PPi release and before dNTP binding steps (Figure 1D). The results from the fits rendered an associated average free energy change for translocation, Δ*G*_trans_ = −0.46*k*_B_*T*, and an effective translocation step size, *δ* = 0.40 nm. These results support an energy landscape for translocation where the polymerase-DNA complex could thermally diffuse between pre- and post- translocated states separated by a distance equivalent to the mean rise per base found in B-DNA, spending ∼1.6 more time in the post-translocated state (Figure 5). The different distances associated with the forward and backward translocation rates, *d*_T_ = 0.35 nm and *d_−_*_T_ = 0.05 nm, respectively, indicate that the transition state barrier is very close to post-translocation state (Figure 5). The effect of opposing load is to shift the translocation equilibrium mainly by slowing the forward translocation rate, *k*_T_(0), or the exit from pre- to the post-translocated state. The similarity we found between the force sensitive forward translocation rate and the rate limiting step of the reaction, *k*_cat_ [*k*_T_(0) is only five times faster than *k*_cat_, Table 1], explains naturally the marked sensitivity of replication velocity to load even at saturated dNTP concentrations (Figure 3A). Therefore, the force-dependent translocation and catalysis control the overall replication velocity and the force–velocity relationship. A similar observation has been recently reported from single molecule mechano-chemical studies of the RNA polymerase II (35). We note that the activity of the Phi29 DNAP is not affected by mechanical tension below ∼20 pN applied longitudinally to the DNA (41–43). This observation indicates that DNA mechanical tension, which is expected to build on the DNA as load is increased on the replicative complex, cannot be responsible for the velocity changes measured even at the smallest aiding or opposing forces. Additional force dependencies on different steps of the replication cycle were not required to fit the experimental data (Supplementary Data), arguing against other possible additional effects of load on the nucleotide incorporation cycle or on the protein activity.

Our data showed that the higher the dNTP concentration, the higher the force required to disrupt or detach the protein-DNA interactions (Figure 3D). Interestingly, for all dNTP concentrations detachments occur when the average replication velocity reaches *v* ∼7 nt/s (Figure 3D inset). In order to better understand the competition between the external load, dNTP binding and protein–DNA detachment we used *v* ∼7 nt/s to estimate, according to Model 3, the probabilities of occupancy of the dNTP/PPi-free (*M*_free_) and dNTP/PPi-bound (*M*_bound_) states right before detachment, obtaining for *M*_free_ ∼94% and for *M*_bound_ ∼6% (Supplementary Data and Supplementary Figure S9B). These results indicate that the dNTP/PPi-bound state is almost as stable under application of opposing loads as the dNTP/PPi-free state. However, for all dNTP concentrations, conditions favoring the occupancy of the dNTP/PPi-free state for times longer than ∼1/7 s promote the load induced detachment of the protein-DNA interactions. But what is the underlying mechanism inducing the detachment? One possible explanation is that the rupture events could be triggered by the intramolecular kinetic partitioning of the primer from the polymerization to the exonucleolysis sites, which has been recently shown to occur from the dNTP/PPi-free state (63). This reaction is known to transiently decrease the number of DNA–protein contacts (43,64–68), increasing in this way the chances of a load-induced protein–DNA detachment. This possibility, in turn, would explain the absence of processive exonuclease measurements in our experiments.

Remarkably, at saturated dNTP concentrations the replication reaction proceeds against surprisingly large opposing loads (up to 50 pN). In other words, the polymerase performs as much as 17 pN nm (∼4 k_B_T) of work per dNTP hydrolysis. According to our data, under these conditions (high load and saturated dNTP concentrations), replication is still possible because the polymerase visits the post-translocated state with a rate of *k*_T_ (50 pN) ∼7 nt/s and the rate of dNTP binding following translocation (i.e: *k*_on_ × [200 μM] > 5 s^−1^ μM^−1^ × 200 μM = 1000 s ^-1^), which is faster than the backwards translocation rate, rapidly stabilizes the DNAP complex into the post-translocated dNTP-bound state, allowing the completion of the replication cycle (Supplementary Figure S9A). Altogether, our data indicate that the work generated by the DNAP is a consequence of the remarkable strength of the protein–DNA interactions against opposing loads (both in the dNTP/PPi -bound and -free states) and also of an indirect effect of dNTP binding, which allows the completion of the nucleotide incorporation cycle and in turn, prevents the kinetic partitioning of the primer strand to the exonucleolysis site from the dNTP/PPi-free conformation.

Our data support a two-state Brownian motor model (69–73) in which the work generated by the polymerase is a direct consequence of the alternation between a dNTP/PPi-free state, where the protein can thermally diffuse between the pre- and post-translocated states, and a dNTP/PPi-bound complex stably bound (pinned) at the post-translocated state. In such a model (and in accordance with previous studies on Family A and B DNA polymerases), in the dNTP/PPi-free state the DNA can freely diffuse in and out of the polymerase active site faster than the relatively slow fingers opening transition that probably accompanies this step (22,54). Thus, bulky tyrosine residues conserved at the base of the fingers subdomain could probe the environment at the active site and pack in front of the newly formed base pair when space allows favoring the post-translocated state (21,54). This state will be further stabilized by binding of the correct incoming dNTP, which in turn would prevent the kinetic partitioning of the primer strand to the exonucleolysis site from the dNTP/PPi-free conformation.

In summary, we have showed that a thermal ratchet mechanism can explain the coupling between chemical catalysis and mechanical translocation of replicative DNA polymerases during processive DNA replication. Furthermore, this mechanism can be efficiently used to generate work against large opposing loads and may play a role in coordinating the synthetic and degradative activities of the protein. In this model, there is no need for a force generating step to directly power translocation; the force generated by the DNAP is an indirect effect of dNTP binding. These results are consistent with recent evidence from bulk and single molecule studies for a growing number of RNA polymerases supporting similar mechanisms (30–35,74–75). Therefore, it is tempting to speculate that a loose mechano-chemical coupling may be a general mechanism for nucleic acid polymerases involved in nucleic acid metabolism.

## SUPPLEMENTARY DATA

Supplementary Data are available at NAR Online.

SUPPLEMENTARY DATA
