# Supplementary material for: Mechano-chemical kinetics of DNA replication: identification of the translocation step of a replicative DNA polymerase
Source: Nucleic Acids Res. 2015 Mar 23;43(7):3643–52. doi: 10.1093/nar/gkv204 (PMC4402526; doi:10.1093/nar/gkv204)
Supplement: SUPPLEMENTARY DATA [file supp_43_7_3643__index.html]

Mechano-chemical kinetics of DNA replication: identification of the translocation step of a replicative DNA polymerase — Mechano-chemical kinetics of DNA replication: identification of the translocation step of a replicative DNA polymerase — SUPPLEMENTARY DATA 

# Mechano-chemical kinetics of DNA replication: identification of the translocation step of a replicative DNA polymerase

## SUPPLEMENTARY DATA

**Files in this Data Supplement:**

- SUPPLEMENTARY DATA
