# Supplementary material for: Mechano-chemical kinetics of DNA replication: identification of the translocation step of a replicative DNA polymerase
Source: Nucleic Acids Res. 2015 Mar 23;43(7):3643–52. doi: 10.1093/nar/gkv204 (PMC4402526; doi:10.1093/nar/gkv204)
Supplement: SUPPLEMENTARY DATA [file supp_gkv204_nar-03631-f-2014-File003.pdf]

Supplementary Data for:

Mechano-chemical kinetics of DNA replication: identification of the translocation step of a replicative DNA polymerase

José A. Morin[1†], Francisco J. Cao[2], José M. Lázaro[3], J. Ricardo Arias-Gonzalez[4], José M. Valpuesta[5], José L. Carrascosa[4,5], Margarita Salas[3] & Borja Ibarra[4,*]

[1]Instituto Madrileño de Estudios Avanzados en Nanociencia, IMDEA Nanociencia. 28049 Madrid, Spain.

[2]Departamento Física Atómica, Molecular y Nuclear. Universidad Complutense. 28040 Madrid, Spain.

[3]Centro de Biología Molecular 'Severo Ochoa' (CSIC-UAM). Universidad Autónoma, Cantoblanco. 28049 Madrid, Spain.

[4]Instituto Madrileño de Estudios Avanzados en Nanociencia (IMDEA Nanociencia) & CNB-CSIC-IMDEA Nanociencia Associated Unit "Unidad de Nanobiotecnología". 28049 Madrid, Spain

[5]Centro Nacional de Biotecnología (CNB-CSIC). Madrid, 28049, Spain.

[†]Present Address: Max-Planck-Institute for the Physics of Complex Systems. Nöthnitzer Strasse 38. 01187 Dresden. Germany

[*]To whom correspondence should be addressed:

Tel: 34 91 2998863

Fax: 34 91 2998725

borja.ibarra@imdea.org

Mechano-chemical models

To determine the location of the translocation step within the replication cycle, we initially considered a minimal nucleotide incorporation cycle where the activation of the ternary complex and the fast following chemical steps were grouped within a single rate limiting step (Figure 1A). Since no significant conformational changes within the polymerase-DNA complex occur during these steps translocation is not expected to occur concomitant to the rate-limiting step of the reaction. Assuming a single force dependent state, or in other words, assuming that translocation is associated with only one step of the cycle, three alternative general models could explain the coupling mechanism between the chemical and mechanical steps during the nucleotide incorporation cycle: In Model 1 (Figure 1B), translocation is power-stroked by dNTP binding, in Model 2 (Figure 1C) translocation is power-stroked by PPi release and in Model 3 (Figure 1D), translocation occurs by thermal diffusion between PPi-free and dNTP-free states. We assumed that the forward and reverse rates of the translocation step present an Arrhenius-like dependence on force

$$k_i(F) = k_i(0)e^{\frac{F \cdot d_i}{k_B T}} \ (eq. 1),$$

where *F* is the applied load, k_B is Boltzmann's constant, *T* is the temperature, and d_i is the effective distance over which the applied load acts on translocation (1).

Three states power-stroke models (Model 1 and Model 2)

The power stroke translocation mechanisms, Model 1 and Model 2, differ in the step of the reaction that propels translocation, dNTP binding and PPi release, respectively. For both mechanisms the minimal kinetic mechanism can be represented with a three state model as

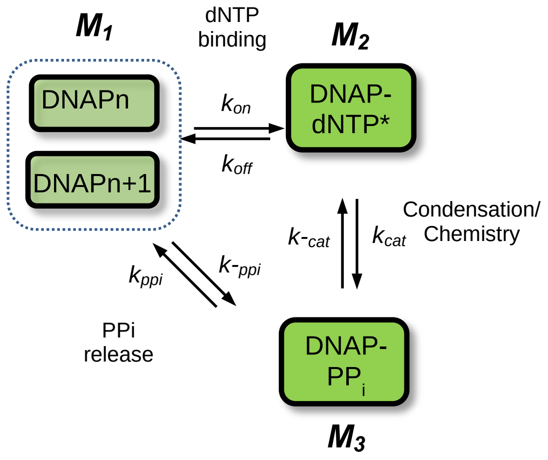

In both cases DNAPn+1 and DNAPn correspond to the same state of the polymerase-DNA complex but displaced by one base-pair distance along the DNA track, δ=0.34 nm. The evolution equations for the occupation probabilities M_i are (2)

$$\frac{dM_1}{dt} = -(k_{-ppi}D + k_{on}T)M_1 + k_{off}M_2 + k_{ppi}M_3$$
$$\frac{dM_2}{dt} = k_{on}TM_1 - (k_{off} + k_{cat})M_2 + k_{-cat}M_3$$
$$\frac{dM_3}{dt} = k_{cat}M_2 - (k_{-cat} + k_{ppi})M_3 + k_{-ppi}DM_1$$

and the conservation condition $M_1 + M_2 + M_3 = 1$ is verified. In the steady state, all the derivatives of the occupation probabilities M_i are zero. T stands for the concentration of nucleotides and D for the concentration of inorganic pyrophosphate, PPi. In particular, when the PPi concentration is negligible, *i.e.* $D \simeq 0$, the polymerization velocity takes the Michaelis Menten (MM) form

$$v = V_{\max}\frac{[dNTP]}{K_M + [dNTP]} \quad (eq.2),$$

where the MM parameters, V_{max} and K_M, (or their inverses) are given in terms of the rates of the reaction (see equations 3 and 4 below). We note that throughout this work we have used the inverses of V_{max} (maximum velocity at saturated dNTP concentrations) and $k_b = V_{\max}/K_M$ (effective rate of dNTP binding), because they

provide more apparent expressions with simpler relations among characteristic times (inverse of the rates) and the processes involved during the cycle. These simple relations provide a straightforward interpretation of the observations and their implications (see below).

The observed force dependency of $1/V_{max}$ rules out Model 1.

According to Model 1 (Figure 1B), translocation is driven by the nucleotide binding reaction. In this case, load opposing translocation would affect specifically the nucleotide binding and/or unbinding rates, $k_{on}[dNTP](F)$ and $k_{off}(F)$. A direct consequence of this model is that the replication velocity at saturated dNTP concentrations, V_{max}, should not depend on force (1-4). This is because V_{max} (or $1/V_{max}$, see equation 3) does not depend on $k_{on}(F)[dNTP]$ and $k_{off}(F)$, which are the only force dependent rates of the cycle in Model 1

$$\frac{1}{V_{max}} = \frac{1}{k_{cat}}\left(1 + \frac{k_{-cat}}{k_{ppi}}\right) + \frac{1}{k_{ppi}} \ (eq. 3).$$

Then, a model proposing that translocation is driven by nucleotide binding, such as Model 1, implies that velocity at saturated dNTP concentrations should be force independent. The observed force dependency of $1/V_{max}$ argues directly against this mechanism (Figure 4B).

The observed force dependency of $1/k_b(F)$ rules out Model 2.

In Model 2 (Figure 1C), translocation occurs during PPi release, being $k_{ppi}(F)$ the only force dependent rate of the nucleotide incorporation cycle. According to this model the inverse of $k_b(F)$ can be written as

$$\frac{1}{k_b(F)} = \frac{K_{\mathrm{M}}(F)}{V_{max}(F)} = \frac{1}{k_{on}}\left[1 + \frac{k_{off}}{k_{cat}}\left(1 + \frac{k_{-cat}}{k_{ppi}(F)}\right)\right] (eq. 4).$$

This model can be excluded if one of the following conditions are verified

$$\frac{k_{off}}{k_{cat}} \cdot \frac{k_{-cat}}{k_{ppi(F)}} \ll 1 \text{ or } \frac{k_{-cat}}{k_{ppi(F)}} \ll 1$$

Kinetics studies of Family A and B DNAPs showed that k_{ppi} is typically ~10^3 times faster than the reverse of the rate limiting step of the reaction, k_{-cat}, implying that $\frac{k_{-cat}}{k_{ppi(F)}} \ll 1$ (5-11) and therefore, $1/k_b$ should be largely independent on force (considering d_i ~0.34 nm). This prediction contrasts with our data showing a load dependency of $1/k_b$ (Figure 4D), arguing against a direct connection between the PPi release step and mechanical translocation.

Altogether our data argue against models where translocation is power-stroked by dNTP binding or PPi release.

Exclusion of alternative 3-state models

We checked the compatibility of the experimental data with an alternative model considering that translocation occurs during the step located between the dNTP-bound and PPi-bound states. As we did for the other 3 models discussed in the manuscript, we considered that this step comprises the rate-limiting activation of the ternary complex and the following rapid chemical steps (references 5 and 10 to 20, main text). In this case, the rate-limiting step was force dependent and their forward and backward rates were defined by $k_{cat}(F)$ and $k_{-cat}(F)$, respectively. Also, as in the other models and according to our data, the PPi release step was considered largely irreversible (k_{-ppi} ~0 s^{-1}, Supplementary Figure S3).

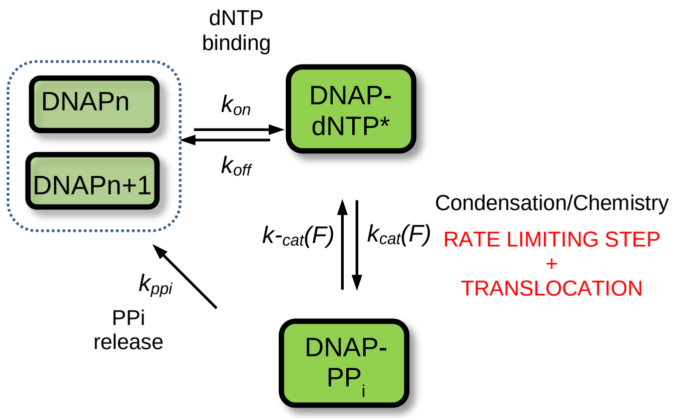

The kinetic expressions for the Michaelis-Menten parameters V_{max}, and K_M derived from this model allowed initially a force dependent behavior for both *1/*V_{max}*(F)* and *1/*k_b*(F)*, since

$$\frac{1}{V_{max}(F)} = \frac{1}{k_{ppi}} + \frac{1}{k_{cat}(F)}\left(1 + \frac{k_{-cat}(F)}{k_{ppi}}\right)\ (eq.5)$$

$$\frac{1}{k_b(F)} = \frac{K_{\mathrm{M}}(F)}{V_{max}(F)} = \frac{1}{k_{on}}\left[1 + \frac{k_{off}}{k_{cat}(F)}\left(1 + \frac{k_{-cat}(F)}{k_{ppi}}\right)\right]\ (eq.6)$$

In order to test the validity of this new model to explain the data, we used the same approximation described in the main text to test the four-state Brownian Ratchet model (Model 3). In this way, a direct comparison between the two models can be done. Therefore, the above expressions for 1/V_{max}*(F)* and 1/k_b*(F)* (equation 5 and 6, respectively) were recast as the sum of a force independent and force dependent terms

$$\frac{1}{V_{max}(F)} = a + b \cdot e^{F \cdot d_b/(k_B T)}\ (eq.7),$$

$$\frac{1}{k_b(F)} = \frac{K_M(F)}{V_{max}(F)} = r + s \cdot e^{F \cdot d_s/(k_B T)}\ \ (eq.8),$$

where the coefficients *a, b, r, s*, are given in terms of the rates of the cycle and, the coefficients d_b and d_s define the force dependency or effective distance over which force acts on translocation, δ. As described in the main text, we included equations 7 and 8 in the Michaelis-Menten velocity definition (equation 2) to obtain *v(F, [dNTP])* and used it to fit simultaneously the force-velocity relationships for all dNTP concentrations. The results of the fits yielded the values of the coefficients *a, b, r, s,* d_b and d_s shown in the Table 1 in the main text.

According to the above relationships it can be shown that $a \sim \frac{1}{k_{ppi}}$. Therefore, since the main contribution to $1/V_{max}$ at zero force comes from the value of the parameter *a* (*a* >

b, Table 1), in this new model the rate of pyrophosphate release, k_{ppi}, must correspond to the rate limiting step of the reaction at saturated dNTP concentrations and presents a value of ~120 s^{-1} (k_{ppi} = *1/a* = 1/0.0085). This result contrasts with steady state and pre-steady state kinetic studies for several DNAPs showing that the PPi release step, k_{ppi}, is one of the fastest rates of the nucleotide incorporation cycle, $k_{ppi} = 10^3$-10^4 s^{-1}, and in any case is rate-limiting (see references 14, 16, 21, 58, 59, 60 and 61 in the main text). In turn, the relationships between the coefficients and the rates also imply that the step comprising k_{cat} cannot be the rate limiting step of the cycle, which again contrasts with many experimental evidences supporting the nucleotide condensation rate as the rate-limiting step of the reaction (references 5 and 10 to 20 in the main text).

In summary, although an alternative kinetic model where translocation occur between dNTP and PPi states may initially explain the force dependence of the experimental data, the results of this model are not supported by multiple experimental evidences showing that:

1. Ternary replication complexes of different polymerases have the same conformations in both, the incoming dNTP and product, PPi, complexes (references 2, 4, 21, 24, and 54 in the manuscript). Structural studies show that the newly incorporated nucleotide occupies the insertion site in both the dNTP-bound and PPi-bound states. These data therefore, clearly indicates that the reactions occurring during these two states (step 2 in our models) are not compatible with the relative translocation of the polymerase DNA complex, which should involve a conformational change or a relative displacement of 0.34 nm between the polymerase and the DNA. Based on these arguments, we did not initially considered a model where translocation occurs during the transition between dNTP-bound and PPi-bound states.

2. Pre-steady-state kinetic studies of several DNA polymerases indicate that after nucleotide binding (and fingers closure), unspecified subtle non-covalent

transformations in the active site activate the ternary complex to form an active site poised for catalysis. This process, also called 'nucleotide condensation', was found to be the rate-limiting step of the nucleotide incorporation cycle (references 5 and 10 to 20 in the main text). These studies are not compatible with the results of the model shown above.

3. The PPi release rate, k_{ppi}, is probably the fastest rate of the nucleotide incorporation cycle, k_{ppi} =10^3-10^4 s^{-1} and to date, it has not been found to be the rate-limiting step on the reaction for any replicative polymerase (references 14, 16, 21, 58, 59, 60 and 61 in the main text). This evidence argues against this alternative model, which requires k_{ppi} to be the rate limiting step of the reaction.

We note that, when translocation is considered to occur associated with a single step of the nucleotide incorporation cycle, the experimental data is not compatible with any model (3 and/or 4 states) where the translocation step is also the rate-limiting step of the reaction. This is because the main contribution to $1/V_{max}$ at zero force comes from the value of the parameter *a,* which does not depend on force (and therefore, is not associated with translocation).

Four states Brownian ratchet mechanism (Model 3)

The Brownian ratchet mechanism, Model 3, considers that translocation occurs by thermal diffusion after PPi release and before dNTP binding, and requires the inclusion of an additional state after PPi release (see below and Figure 1D). In this case, the force dependent rates of the reaction are the forward and backward translocation rates, $k_T(F)$ and $k_{-T}(F)$ respectively.

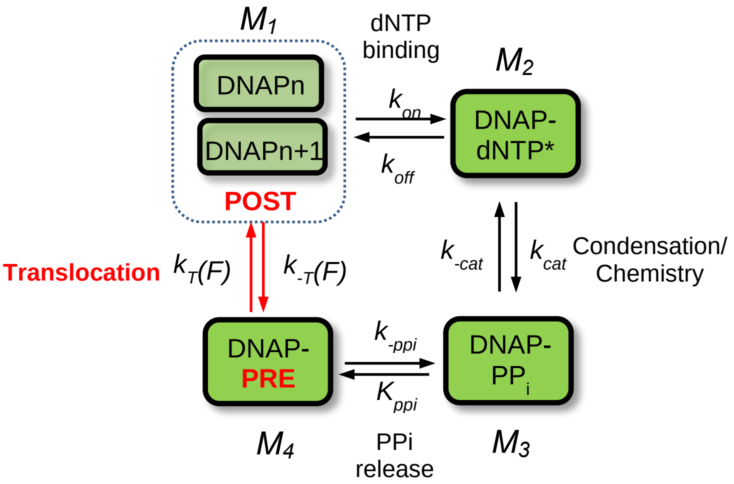

The evolution equations for the occupation probabilities M_i of this four states model are:

$$\frac{dM_1}{dt} = -(k_{-T} + k_{on}T)M_1 + k_{off}M_2 + k_T M_4$$
$$\frac{dM_2}{dt} = k_{on}TM_1 - (k_{off} + k_{cat})M_2 + k_{-cat}M_3$$
$$\frac{dM_3}{dt} = k_{cat}M_2 - (k_{-cat} + k_{ppi})M_3 + k_{-ppi}DM_4$$
$$\frac{dM_4}{dt} = k_{-T}M_1 + k_{ppi}M_3 - (k_{-ppi}D + k_T)M_4$$

and the conservation condition $M_1 + M_2 + M_3 + M_4 = 1$ is verified. In the steady state all the derivatives of the occupation probabilities M_i are zero. Again, T stands for nucleotide concentration and D for product (PPi) concentration.

In contrast to the other two models, the kinetic expressions for the Michaelis-Menten parameters V_{max}, and K_M derived from the Brownian ratchet model (Model 3) allow initially a force dependent behavior for both $1/V_{max}(F)$ and $1/k_b(F)$

$$\frac{1}{V_{max}(F)} = \frac{1}{k_{cat}}\left(1 + \frac{k_{-cat}}{k_{ppi}}\right) + \frac{1}{k_{ppi}} + \frac{1}{k_T(F)} \; (eq.9),$$

$$\frac{1}{k_b(F)} = \frac{K_M(F)}{V_{max}(F)} = \frac{1}{k_{on}}\left[1 + \frac{k_{off}}{k_{cat}}\left(1 + \frac{k_{-cat}}{k_{ppi}}\right)\right]\left(1 + \frac{k_{-T}(F)}{k_T(F)}\right) \; (eq.10).$$

Each of these expressions can be recast as the sum of a force independent and a force dependent term

$$\frac{1}{V_{max}(F)} = a + b \cdot e^{F \cdot d_b/(k_B T)} \text{ , (eq. 11),}$$

$$\frac{1}{k_b(F)} = \frac{K_M(F)}{V_{max}(F)} = r + s \cdot e^{F \cdot d_s/(k_B T)}, (eq. 12),$$

where the coefficients *a, b, r, s*, are given in terms of the rates of the cycle and, the coefficients d_b and d_s define the force dependency or effective distance over which force acts on translocation, δ.

In order to test the validity of this model, we included equations 11 and 12 in the Michaelis-Menten velocity definition (equation 2) to obtain *v(F, [dNTP])* and used it to fit the force-velocity relationships for all dNTP concentrations (Figures 3A, 3B and 3C). After minimizing the sum of the squared errors, we obtained from the fits the values for the 6 parameters in equations 11 and 12.

6-param	a	b	d_b	r	s	d_s
	0.0084 s	0.0015 s	0.35 nm	0.19 s	0.12 s	0.40 nm

Table S1. Best fit values of the parameters in eq. 11 and eq. 12.

Importantly, these values also support the observed force dependency for $1/V_{max}(F)$, $K_M(F)$ and $1/k_b(F)$ (Figures 4B, 4C and 4D, respectively). In addition, they predict the observed dNTP concentration dependence of the detachment load (black solid line in Figure 3D).

<u>Detachment load determination</u>

Briefly, we found that the detachment load (*F*) as a function of dNTP concentration (*T*) is accurately predicted by *v(T,F)* = 7 nt/s, with *v(T,F)* given by a Michaelis-Menten expression for velocity where $1/V_{max}$ and K_M/V_{max} expressions were substituted by the

above equations 11 and 12, respectively. We recall that 7nt/s correspond to the weighted average replication velocity right before detachment (Figure 3D Inset). These predictions highlight the consistency of the Brownian ratchet model (Model 3) to explain the whole set of experimental data.

Determination of rates

We note that there is a direct relationship between the parameters obtained from the fits (equations 11 and 12, Table S1) and several of the rates and conformational changes of the nucleotide incorporation cycle. We used these relationships for the following calculations:

1.- Calculation of rates and conformational changes related to translocation:

The relations between the free coefficients and the forward (k_T) and backward (k_{-T}) translocation rates are the following:

$$b = \frac{1}{k_T(0)};\quad s = \frac{k_{-T}(0)}{k_T(0)} \cdot r$$

These relations imply $k_T(0) = \frac{1}{b} = 670\ s^{-1}$; $\frac{k_{-T}(0)}{k_T(0)} = \frac{s}{r} = 0.63$, and therefore, $k_{-T}(0) = 420\ s^{-1}$ (Table S1). Therefore, $K_{\delta}(0) = \frac{k_T(0)}{k_{-T}(0)} = 1.59$ and the average free energy of the translocation step is $\Delta G_{trans} = -\ln K_{\delta}\ k_B T \sim -0.46\ k_B T$. This result points out that the translocation reaction is displaced towards the post-translocated state.

The coefficients d_b and d_s are directly related with the characteristic distances from the pre-translocation position to the transition state (d_T), and from the transition state to the post-translocation state (d_{-T}) as follows:

$$d_b = d_T \text{ and } d_s = d_T + d_{-T}.$$

Therefore, $d_T = d_b = 0.35\,nm$ and $d_{-T} = d_s - d_T = 0.05\,nm$, which gives a translocation step size d_s= d_T + d_{-T} ~ 0.4 nm. This distance is compatible with the expected distance between the pre- and post-translocated states, δ~ 0.34 nm (12-14).

Importantly, the values obtained for the rates and conformational changes associated with translocation are consistent with the Brownian Ratchet mechanism, Model 3, where translocation can occur by thermal diffusion of the polymerase-DNA complex between pre- and post- translocated states separated by a distance equivalent to the mean rise per base found in B-DNA.

2. Calculation of the nucleotide condensation and catalysis rate, k_{cat}:

The value of k_{cat} can be derived from the relation $a = \frac{1}{k_{cat}}\left(1 + \frac{k_{-cat}}{k_{ppi}}\right) + \frac{1}{k_{ppi}}$. Since the rate of PPi release, k_{ppi}, is very fast (~10^3-10^4 s^{-1}) and 10^3 times faster than k_{-cat} (5-11), the ratios $\frac{k_{-cat}}{k_{ppi}}$ and $\frac{1}{k_{ppi}}$ are both much smaller than 1, implying that $k_{cat} \sim \frac{1}{a} = 120\ s^{-1}$. Note that the main contribution to $1/V_{max}$ at zero force comes from a (since $a > b$), pointing out that k_{cat} corresponds to the rate limiting step of the reaction at saturated dNTP concentrations. Interestingly, the results from the fits directly showed that the rate limiting step of the nucleotide incorporation cycle does not depend on force and therefore, it is not associated with translocation. This result is in agreement with our initial assumptions, which were based on previous kinetic and structural studies on several DNAPs (6,14-17).

3. Calculation of the nucleotide binding rate, k_{on}:

The value of k_{on} can be derived from the relation $r = \frac{1}{k_{on}}\left[1 + \frac{k_{off}}{k_{cat}}\left(1 + \frac{k_{-cat}}{k_{ppi}}\right)\right]$. As mention before, k_{ppi} is ~10^3 times faster than k_{-cat} (5-11), implying that the ratio $\frac{k_{-cat}}{k_{ppi}} \ll 1$. Therefore, the coefficient r can be written as follows:

$$r = \frac{1}{k_{on}} + \frac{k_{off}}{k_{on}}\frac{1}{k_{cat}} = \frac{1}{k_{on}} + \frac{1}{K_1}\frac{1}{k_{cat}}$$

$K_1 = \frac{k_{on}}{k_{off}}$ is the dNTP dissociation constant, which for the Phi29 DNAP has been recently determined as K_1 = 1.4 μM (18) and as shown above, $\frac{1}{k_{cat}} \sim a$ (a = 0.0084, Table S1). Therefore, $k_{on} \sim 5.43\, s^{-1}\mu M^{-1}$, which is consistent with the value of the dGTP binding rate (k_{on} ~17 μM^{-1} s^{-1}) recently determined for the Phi29 DNAP (19).

<u>Calculation of the probabilities of occupancy of the different states of the Brownian Ratchet model (Model 3)</u>

The occupation probabilities (M_i) of each state of the Brownian ratchet model (Model 3) in the stationary state are given by

$$\begin{aligned} M_1 = {} & (k_{-cat}k_F(F)k_{off} + k_{ppi}k_{off}k_T(F) + k_{ppi}k_{cat}k_T(F))/(k_{-cat}k_T(F)k_{on}T \\ & + k_{-cat}k_{off}k_{-T}(F) + k_{-cat}k_T(F)k_{off} + k_T(F)k_{ppi}k_{on}T \\ & + k_T(F)k_{cat}k_{on}T + k_{ppi}k_{cat}k_{on}T + k_{ppi}k_{cat}k_{-T}(F) + k_{ppi}k_{off}k_T(F) \\ & + k_{ppi}k_{off}k_T(F) + k_{ppi}k_{off}k_{-T}(F) + k_{ppi}k_{cat}k_T(F)) \end{aligned}$$

$$\begin{aligned} M_2 = {} & (k_T(F)k_{ppi}k_{on}T + k_{-cat}k_T(F)k_{on}T)/(k_{-cat}Tk_{on}T + k_{-cat}k_{off}k_{-T}(F) \\ & + k_{-cat}k_T(F)k_{off} + k_Tk_{ppi}k_{on}T + k_T(F)k_{cat}k_{on}T + k_{ppi}k_{cat}k_{on}T \\ & + k_{ppi}k_{cat}k_{-T}(F) + k_{ppi}k_{off}k_T(F) + k_{ppi}k_{off}k_{-T}(F) \\ & + k_{ppi}k_{cat}k_T(F)) \end{aligned}$$

$$M_3 = (k_T(F)k_{cat}k_{on}T)/(k_{-cat}k_T(F)k_{on}T + k_{-cat}k_{off}k_{-T}(F) + k_{-cat}k_T(F)k_{off} + k_T(F)k_{ppi}k_{on}T + k_T(F)k_{cat}k_{on}T + k_{ppi}k_{cat}k_{on}T + k_{ppi}k_{cat}k_{-T}(F) + k_{ppi}k_{off}k_T(F) + k_{ppi}k_{off}k_{-T}(F) + k_{pi}k_{cat}k_T(F))$$

$$M_4 = (k_{ppi}k_{cat}k_{on}T + k_{-cat}k_{off}k_{-T}(F) + k_{ppi}k_{off}k_{-T}(F) + k_{ppi}k_{cat}k_{-T}(F))/(k_{-cat}k_T(F)k_{on}T + k_{-cat}k_{off}k_{-T}(F) + k_{-cat}k_T(F)k_{off} + k_T(F)k_{ppi}k_{on}T + k_T(F)k_{cat}k_{on}T + k_{ppi}k_{cat}k_{on}T + k_{ppi}k_{cat}k_{-T}(F) + k_{ppi}k_{off}k_T(F) + k_{ppi}k_{off}k_{-T}(F) + k_{ppi}k_{cat}k_T(F))$$

T refers to nucleotide concentration and k_i and k_{-i} correspond to the forward and backward rates, respectively, of the different step of the Brownian ratchet model (Model 3) described before and in Figure 1D. To estimate the load dependent occupancy probability (M_i) of each state of the replication cycle, we used the values of the rates and force dependencies obtained in our work (Table 1 in main text) plus the following values for the rates that cannot be calculated with our model:

-k_{off}, the dNTP unbinding rate, has been recently reported for the Phi29 DNAp, k_{off}= 30 s^{-1} (19).

-Based on pre-steady state and steady state kinetic studies on different replicative polymerases (5-11), we considered:

k_{-cat}, the reverse of the rate-limiting step, as k_{-cat} ~30 s^{-1} .

k_{ppi}, the rate of the PPi release step, as k_{ppi}~ 10^3 s^{-1}.

The occupancy probabilities versus load estimated for each state of the replication cycle are shown in Supplementary Figure S9A.

Probability of occupancy of the dNTP/PPi-bound and dNTP/PPi-free states at conditions promoting detachment

We estimate the occupation numbers of the dNTP/PPi-bound and dNTP/PPi-free states previous to detachment in the following way. According to our data, close to the detachment conditions the polymerase passes through dNTP-bound state 7 times per second (v~ 7 nt/s, Figure 3D Inset). If M_2 is the occupation probability of the dNTP-bound state, the relation $M_2 * k_{cat} \sim v$ gives that $M_2 \sim v/k_{cat}$. Thus, right before detachment $M_2 \sim$ 7/120 =0.06 =6%. As the PPi-bound state is irreversible and $k_{ppi} \gg k_{-cat}$ (5-11), we have that the occupation probability of the PPi-bound state, M_3, is negligible and $M_3 \ll M_2$. Thus, under conditions promoting detachment, the probability to find the polymerase in a dNTP/PPi-free state is 94%, strongly suggesting that detachments occur from the dNTP/PPi-free state. Supplementary Figure S9B shows the effect of load on the probability of occupancy of the dNTP/PPi-free state.

Exclusion of alternative 4-state models

As mention before, we initially excluded the possibility that translocation may occur associated to the rate limiting step. This assumption was made based on extensive biochemical and structural data showing that no significant conformational changes within the polymerase-DNA complex occur during this reaction (6,14-17). Interestingly, as shown before, the results from the fits directly corroborated that the rate limiting step of the nucleotide incorporation cycle does not depend on force and therefore, it is not

associated with translocation. Please, see the previous section, '*Exclusion of alternative 3-state models'*, for a detailed description of this point.

Additionally, we checked whether alternative four-state models considering that translocation occurs during dNTP binding or PPi release steps are valid to explain the data.

Translocation cannot occur during the nucleotide binding reaction. In this case, translocation would be driven by the nucleotide binding reaction and therefore, load opposing translocation would affect specifically the nucleotide binding and/or unbinding rates, $k_{on}[dNTP](F)$ and $k_{off}(F)$, respectively. However, as shown in equation 9, the replication velocity at saturated dNTP concentrations, $1/V_{max}$, is independent of $k_{on}[dNTP](F)$ and $k_{off}(F)$, implying a load independent $1/V_{max}$, which contradicts our measurements. This possibility is equivalent to the three-state power stroke Model 1 described above.

The PPi release process cannot be directly associated with translocation if $\frac{k_{-cat}}{k_{ppi}(F)} \ll 1$. According to equation 10, these conditions would imply that $1/k_b$ should be force independent, which is not reflected in our data. As mentioned before, kinetics studies on Family A and B DNAPs showed that k_{ppi}>>k_{-cat} (5-11), implying that $\frac{k_{-cat}}{k_{ppi}(F)} \ll 1$ and therefore, arguing against the direct coupling between PPi and translocation. This possibility is equivalent to the power stroke Model 2.

Additional force dependencies are not required to fit the data

Based on kinetic and structural studies on different DNAPs, the models proposed in this work assume that translocation is associated to a single step of the nucleotide incorporation cycle. Since load is expected to affect specifically the step of the cycle related to motion, all the models proposed here present a single load dependent step.

However, alternatively one could consider either that translocation is distributed among several steps of the cycle or that load modifies, besides the translocation step, other steps of the nucleotide incorporation reaction, ie: by affecting the polymerase structure or the polymerase-DNA interactions. In these cases more than one step of the cycle would depend on force. We checked these possibilities by testing four-state models with several force dependent steps. We found that, although these models involved more parameters, they did not significantly improve the fit to data (not shown). Although the possibility of additional force dependencies cannot be totally excluded with our present analysis of data, these results strongly suggest that a 'simple' four-state model with single force dependent step is good enough to explain the experimental data. According to the results discussed above this translocation step occurs after PPi release and before dNTP binding.

Supplementary Materials and Methods

DNA Templates. We designed the following DNA construct to measure replication against hindering loads (Supplementary Figure S1A). The *pBacgus11* (Novagen) dsDNA vector (8041 bp) was first nicked at position 564 with *NBbvIA* nicking enzyme (New England Biolabs). The nicked vector was subsequently digested at position 315 with the *BstXI* restriction endonuclease (New England Biolabs), which leaves a 4 nucleotide long protruding 3' ends. Then the nicked strand was digested from the 3' to the 5' end (positions 564 to 315) by the *Exonuclease III* (New England Biolabs). The *Exonuclease III* reaction was stopped with EDTA (10 mM) and Proteinase K (0.1 μg/μl) and the DNA vector was linearized with *BamHI*. The final DNA molecule consists of a 3487 bp section with a protruding 249 nucleotide long 3' end. A digoxigenin labeled DNA handle (20) was ligated to the *BamHI* end and a 20-mer oligonucleotide (5'-

GTGTGTGGGTGAAGTCATGC-3') complementary to the protruding 3' end was annealed to act as a primer for DNA replication.

The following DNA construct was designed to apply aiding loads during replication (Supplementary Figure 1B). A 3487 long dsDNA molecule with a 249 nucleotide protruding 3' end was generated as described above. In this case, a 24-mer oligonucleotide (5'-CTAGGTGTGTGGGTGAAGTCATGC-3') was annealed to the protruding 3' end leaving a 4 nucleotide long 5'-overhang (5'-CTAG). The overhand was used to ligate the pUC19 dsDNA vector (2,686 bp) previously digested with *XbaI* and labeled with digoxigenin at one end (20). The dsDNA molecule added upstream the 3' end of the primer provides separation between the beads (~1 μm) and can be used as a handle to apply aiding loads to the polymerase-DNA complex.

Conversion of distance changes to replicated nucleotides. The number of nucleotides incorporated at a particular load was obtained by dividing the measured distance change between the beads by the average distance between single- (for primer extension activity) or doubled-stranded (for strand displacement activity and aiding loads) nucleotides at that load. The extension between dsDNA nucleotides at each force was calculated using the worm-like chain (WLC) model for polymer elasticity with a persistent length of P = 53 nm and stretch modulus S = 1200 pN / nm for dsDNA. The extension between ssDNA nucleotides at each force was calculated, as described elsewhere (20,21), by subtracting the extension of the dsDNA fraction to the final force-extension curve of the hybrid molecule at each force and dividing by the total number of ssDNA nucleotides (Supplementary Figure S4).

Biotin labeling of the Phi29 DNA polymerase. A commercial kit (Avidity) was used to label *in vivo* the amino terminal domain of the Phi29 DNAP with a single biotin. Briefly, the gene containing the Phi29 DNAP sequence was inserted into a pAN vector downstream the peptide sequence GLNDIFEAQKIEWHE (Avitag sequence). The

recombinant protein was expressed in an *E. coli* K12 strain (AVB100) containing the biotin ligase BirA, which binds covalently a single biotin to the Avitag sequence. The polymerase labeled with biotin was purified to homogeneity following standard methods described elsewhere (22). We ran standard bulk biochemical assays to test the polymerization, the exonucleolysis and strand displacement activities of the tagged polymerase. Comparison with the activity of the wild-type polymerase shows that the amino terminal biotin labeling does not significantly alter the main activities of the Phi29 DNAP (Supplementary Figure S2).

Supplementary References:

1. Bustamante, C., Chemla, Y.R., Forde, N.R. and Izhaky, D. (2004) Mechanical Processes in Biochemistry. *Annual Review of Biochemistry*, **73**, 705-748.
2. Keller, D. and Bustamante, C. (2000) The Mechanochemistry of Molecular Motors. *Biophysical Journal*, **78**, 541-556.
3. Thomen, P., Lopez, P.J., Bockelmann, U., Guillerez, J., Dreyfus, M. and Heslot, F. (2008) T7 RNA Polymerase Studied by Force Measurements Varying Cofactor Concentration. *Biophysical Journal*, **95**, 2423-2433.
4. Herbert, K.M., Greenleaf, W.J. and Block, S.M. (2008) Single-Molecule Studies of RNA Polymerase: Motoring Along. *Annual Review of Biochemistry*, **77**, 149-176.
5. Wong, I., Patel, S.S. and Johnson, K.A. (1991) An induced-fit kinetic mechanism for DNA replication fidelity: direct measurement by single-turnover kinetics. *Biochemistry*, **30**, 526-537.
6. Rothwell, P.J. and Waksman, G. (2005) Structure and mechanism of DNA polymerases. *Advances in Protein Chemistry*, **71**, 40.
7. Patel, S.S., Wong, I. and Johnson, K.A. (1991) Pre-steady-state kinetic analysis of processive DNA replication including complete characterization of an exonuclease-deficient mutant. *Biochemistry*, **30**, 511-525.
8. Lowe, L.G. and Guengerich, F.P. (1996) Steady-State and Pre-Steady-State Kinetic Analysis of dNTP Insertion Opposite 8-Oxo-7,8-dihydroguanine by Escherichia coli Polymerases I exo- and II exo- †. *Biochemistry*, **35**, 9840-9849.
9. Kirmizialtin, S., Nguyen, V., Johnson, Kenneth A. and Elber, R. (2012) How Conformational Dynamics of DNA Polymerase Select Correct Substrates: Experiments and Simulations. *Structure*, **20**, 618-627.
10. Joyce, C.M. and Benkovic, S.J. (2004) DNA Polymerase Fidelity: Kinetics, Structure, and Checkpoints†. *Biochemistry*, **43**, 14317-14324.
11. Donlin, M.J., Patel, S.S. and Johnson, K.A. (1991) Kinetic partitioning between the exonuclease and polymerase sites in DNA error correction. *Biochemistry*, **30**, 538-546.
12. Li, Y., Korolev, S. and Waksman, G. (1998) Crystal structures of open and closed forms of binary and ternary complexes of the large fragment of Thermus aquaticus DNA polymerase I: structural basis for nucleotide incorporation. *EMBO J*, **17**, 7514-7525.

13. Abbondanzieri, E.A., Greenleaf, W.J., Shaevitz, J.W., Landick, R. and Block, S.M. (2005) Direct observation of base-pair stepping by RNA polymerase. *Nature*, **438**, 460- 465.
14. Berman, A.J., Kamtekar, S., Goodman, J.L., Lázaro, J.M., de Vega, M., Blanco, L., Salas, M. and Steitz, T.A. (2007) Structures of phi29 DNA polymerase complexed with substrate: the mechanism of translocation in B-family polymerases. *EMBO J*, **26**, 3494-3505.
15. Steitz, T.A. (2006) Visualizing polynucleotide polymerase machines at work. *The EMBO Journal*, **25**, 3458-3468.
16. Nakamura, T., Zhao, Y., Yamagata, Y., Hua, Y.-j. and Yang, W. (2012) Watching DNA polymerase η make a phosphodiester bond. *Nature*, **487**, 196-201.
17. Truniger, V., Lázaro, J.M., Esteban, F.J., Blanco, L. and Salas, M. (2002) A positively charged residue of φ29 DNA polymerase, highly conserved in DNA polymerases from families A and B, is involved in binding the incoming nucleotide. *Nucleic Acids Research*, **30**, 1483-1492.
18. Dahl, J.M., Mai, A.H., Cherf, G.M., Jetha, N.N., Garalde, D.R., Marziali, A., Akeson, M., Wang, H. and Lieberman, K.R. (2012) Direct Observation of Translocation in Individual DNA Polymerase Complexes. *Journal of Biological Chemistry*, **287**, 13407-13421.
19. Lieberman, K.R., Dahl, J.M., Mai, A.H., Cox, A., Akeson, M. and Wang, H. (2013) Kinetic Mechanism of Translocation and dNTP Binding in Individual DNA Polymerase Complexes. *Journal of the American Chemical Society*, **135**, 9149-9155.
20. Ibarra, B., Chemla, Y.R., Plyasunov, S., Smith, S.B., Lázaro, J.M., Salas, M. and Bustamante, C. (2009) Proofreading dynamics of a processive DNA polymerase. *EMBO J*, **28**, 2794-2802.
21. Morin, J.A., Cao, F.J., Lazaro, J.M., Arias-Gonzalez, J.R., Valpuesta, J.M., Carrascosa, J.L., Salas, M. and Ibarra, B. (2012) Active DNA unwinding dynamics during processive DNA replication. *Proceedings of the National Academy of Sciences*, **109**, 8115-8120.
22. Lazaro, J.M., Blanco, L. and Salas, M. (1995) Purification of bacteriophage Phi29 DNA polymerase. *Methods Enzymol* **262**, 42-49.
23. Jahnel, M., Behrndt, M., Jannasch, A., Schäffer, E. and Grill, S.W. (2011) Measuring the complete force field of an optical trap. *Opt. Lett.*, **36**, 1260-1262.

Supplementary Figure Legends.

Supplementary Figure S1. Template DNA. Schematic diagrams of the DNA templates used to monitor single replication events hindered (**A**) or aided (**B**) by load. See supplementary methods for template construction. We took advantage of the great processivity and strong strand displacement activity of the Phi29 DNAP to measure single replication events along thousands of nucleotides. **C)** Schematic diagrams of experimental configuration for application of load opposing (upper panel) or aiding (lower panel) translocation. A single polymerase molecule (blue triangle) is attached to a bead (blue) held on a micropipette and tethered via the upstream DNA template (opposing force) or the downstream DNA template (aiding force) to a bead (grey) in the optical trap. DNA constructs are explained in (A) and (B). In the opposing force geometry, load (F, red arrow) is applied against the translocation direction, while in the aiding geometry, load (F, green arrow) is applied in the same direction of translocation. For explanatory purposes, the pipette and the laser trap are shown aligned along the horizontal axis.

Supplementary Figure S2. Biochemical characterization of the Phi29 DNA polymerase. A) Strand displacement coupled to M13 DNA replication. The reaction mixture contained, in a final volume of 25 µl, 50 mM Tris–HCl (pH 7.5), 1 mM DTT, 4% (v/v) glycerol, 0.1 mg/ml bovine serum albumin, 10 mM $MgCl_2$, 40 µM of each of the four dNTPs, [α-^{32}P]dATP (1 µCi), 4.2 nM singly-primed M13 ssDNA and 16.8 nM either wild type or biotin tagged Phi29 DNA polymerases. After incubation at 25 °C for the indicated times, the reaction was stopped by addition of EDTA to 10 mM and SDS to 0.1% (w/v). After filtration through Sephadex G-50 spin columns, the Čerenkov radiation of the excluded volume was determined to calculate the relative activity values. For size analysis, the labeled DNA was denatured by treatment with 0.7 M

NaOH and subjected to electrophoresis in alkaline 0.7% (w/v) agarose gels and further autoradiography. The indicated migration position of unit-length M13 DNA (7250 bp) was detected by staining with ethidium bromide. **B**) DNA polymerization/exonucleolysis coupled assay. A ^{32}P-labeled dsDNA molecule (15 base pairs) containing a six nucleotide 5′-protruding end was used as the substrate for DNA-dependent DNA polymerization. The incubation mixture contained, in a volume of 12.5 µl, 50 mM Tris–HCl (pH 7.5), 10 mM $MgCl_2$, 1 mM DTT, 4% (v/v) glycerol, 0.1 mg/ml bovine serum albumin, 1.2 nM 5′-labeled dsDNA, 30.3 nM wild type or biotin tagged Phi29 DNA polymerases and the indicated increasing concentrations of the four dNTPs. After incubation at 25 °C for 10 min, the reaction was stopped by adding EDTA to a final concentration of 10 mM. Samples were analyzed by autoradiography after electrophoresis in 20% polyacrylamide gels containing 8 M urea. Polymerization or 3′-5′-exonucleolysis was detected as an increase or decrease, respectively, in the size (15-mer) of the 5′-^{32}P labeled primer.

Supplementary Figure S3. Increasing PPi concentration 1000-fold has no effect on the average replication velocity. To study the process of product (PPi) release, we investigated the effect of PPi concentration on the replication velocity. To minimize the competition of dNTP for binding to the polymerase we further decreased the dNTP concentration to 2 μM and increased the PPi concentration 1000-fold, from 1 μM to 1 mM. The figure shows that similar average replication velocities where found at 1 μM (black squares) and 1 mM (red dots) PPi concentrations at different aiding loads. Error bars represent s.e. Data was collected in Stephan Grill laboratory (MPI-CBG, Dresden) with a dual trap optical tweezers apparatus with an enhanced position resolution at 1 KHz and 28 ± 0.5 ^{0}C (23). 2 μM is the lowest dNTP concentration yielding a good signal/noise ratio.

Supplementary Figure S4. Determination of the ssDNA force-extension curve under the experimental conditions. The extension between single-stranded nucleotides at each force (black dots) was directly obtained by subtracting the extension of the dsDNA fraction at each force to the final force-extension curve of the hybrid molecule and dividing by the total number of ssDNA nucleotides (229 nt). The force extension curve of the dsDNA was calculated using the Worm Like Chain (WLC) model of polymer elasticity with a persistence length, P = 53 nm and stretch module, S = 1200 pN / nm. The average distances per nucleotide as a function of force were calculated from 20 independent molecules. Errors show s.e. Red line shows the change with force of the average distance between single-stranded nucleotides calculated using the WLC model of polymer elasticity with P = 0.75 nm and S = 800 pN / nm. The plot shows the region with major discrepancies between the experimental data and the model. Above 15 pN experimental data are well fitted by the WLC model (not shown).

Supplementary Figure S5. Analysis of pause behaviour. A) At any given force and for all dNTP concentrations the replication velocity histogram typically shows a bimodal distribution: one peak representing an inactive pause state (centred at ~0 nt/s) and the other peak an active moving state (positive velocities) of the protein (see also Figure 2C). The figure shows the velocity distribution corresponding to the average of 7 independent activities taken within the same force range, -15 pN, during strand displacement replication conditions (50 μM dNTPs,). Blue line represents the double Gaussian fit. 'Zero velocity peak', green line: weight= 0.44, mean= 4.18 nt/s and standard deviation (stdv)= 4.58 nt/s. 'Positive velocity peak', red line: weight= 0.53, mean= 25.98 nt/s and stdv= 3.12 nt/s. Velocity points below a threshold (dashed black line) were considered as pause events along the replication run. The threshold was set as the velocity value of two standard deviations below the moving state peak. **B)**

Replicated nucleotides versus time traces showing identified pause events in red (-15 pN, 50 μM dNTPs, strand displacement conditions). For clarity of display only a portion of each run is showed and runs are shifted along the time axis. Pause identification resolution was 0.4-0.8 seconds.

Figures C and D, show the pause behaviour during strand displacement conditions at different loads and dNTP concentrations: 500 μM (blue triangles), 200 μM (red circles), 100 μM (black squares), 50 μM (black empty squares), 10 μM (red empty circles), 5 μM (blue empty triangles). **C)** Pause frequency (pause number / second) versus force. Red line represents the linear fit to the data (slope= -0.001 ± 0.001). **D)** Pause duration (seconds) versus applied load. Red line represents linear fit to the data (slope= -0.009 ± 0.003).

Figures E and F compare the pause behaviour during primer extension and strand displacement replication conditions at different loads and dNTP concentrations. **E)** Pause frequency (number of pauses per second) and **F)** pause duration (seconds) versus force during strand displacement (full circles) and primer extension (empty circles) replication conditions. For Figures E and F, dNTP concentrations are 100 μM (green circles), 50 μM (black circles), 5 μM (blue circles) and red lines represents linear fits to the data (slope in C= 0.004 ± 0.002, slope in D= -0.01 ± 0.00). For all figures error bars show standard error (s.e.).

Together these results indicate that the frequency and duration of pause events do not depend on load, dNTP concentration and replication mode (similar pause behaviour was observed during primer extension and strand displacement replication conditions).

Supplementary Figure S6. Pause events do not affect the force-velocity relationship. The plot shows the effect of load on the average replication velocities with (full circles) and without pauses (empty circles) taken at different dNTP concentrations: 100 μM (green circles), 50 μM (black circles), 5 μM (blue circles). Only data taken during strand displacement conditions is shown. Error bars represent s.e.

Supplementary Figure S7. Primer extension and strand displacement replication rates versus force. Strand displacement (full circles) and primer extension (empty circles) average replication rates (with pauses) plotted against force at different dNTP concentrations: 50 μM (black circles), 10 μM (olive circles), 5 μM (blue circles). The calculation of the number of nucleotides incorporated as a function of time at a particular load for each replication condition is described in the Methods sections (Supplementary Data and main text) and in Supplementary Figure S4. Error bars represent s.e.

No significant differences were found between the force-velocity relationships corresponding to the primer extension and strand displacement replication conditions, suggesting that load does not act on the mechanism used by the polymerase to separate the two strand of the DNA. Previous single molecule manipulation studies in our laboratory, showed that mechanical tension applied on the DNA favours the strand displacement rate but does not affect the the primer extension rate of the Phi29 DNA polymerase (21). The differences between our former and current studies indicate that, load applied directly to the polymerase (current work) and mechanical tension applied to the DNA track (previous work), affect different mechanical coordinates of the reaction (1), modifying the polymerase activities in different ways.

Supplementary Figure S8: Detachment events. A) The plot shows independent replication activities taken in the 'no feedback' mode of operation at 100 (blue), 50 (green) and 10 (red) μM dNTP concentrations. In these experiments, as replication proceeds, opposing load builds up gradually on the polymerase-DNA complex until the attachment between the beads breaks (sudden force drop indicated with a black arrow). Long pauses or reverse exonuclease-like movements were not observed before detachment. Runs were shifted along the time axis for clarity of display. B) Force-jump experiments to estimate the force required to stall the polymerase in the aiding geometry. The figure shows a representative trace of the aiding force-jump experiments. After getting a single attachment, force is initially kept constant at 5 pN. Upon arrival on dNTPs, replication starts; this is detected as an increase in the distance between the beads (green arrows). During active replication, the force on the replication complex was increased in rapid jumps (force jumps, vertical black arrows). In this way, the activity was measured at increasing constant aiding forces on the replication complex (green arrows). The plot shows replication activity at constant aiding loads of 5, 8, 11 and 14 pN. For all dNTP concentrations, detachment of the polymerase-DNA interactions occurred at aiding loads greater than 20 pN (red arrow). C) Maximum aiding loads at which replication can be recorded at different dNTP concentrations. Each dot represents the average of at least 5 independent activities. Red line represent linear fit to the data (slope=0.009 ± 0.001). Error bars correspond to the s.e.m at each force.

Supplementary Figure S9. State occupancy probabilities. A) Probability of occupancy of each state of the nucleotide incorporation cycle according to the Brownian ratchet model (Model 3) as a function of force and dNTP concentrations. Black, red, dark blue, green, pink and light blue lines correspond to 500, 200, 100, 50, 10 and 5 μM dNTP concentrations, respectively. For each plot vertical axis represents

the probability of occupancy and the horizontal axis the applied force. Note the different scale of the plot corresponding to PPi release step. Mainly due to the fast rate of PPi release (k_{ppi} ~10^4 s^{-1}) the PPi-bound state is the less populated condition. The plot shows the relevant scale for this state. All the rate constants are defined in the main and supplementary texts. **B)** Estimation of the probability of occupancy of the dNTP/PPi-free state as a function of force and dNTP concentrations. Black, red, dark blue, green, pink and light blue lines correspond to 500, 200, 100, 50, 10 and 5 μM dNTP concentrations, respectively. According to the Brownian ratchet model, a dNTP/PPi-free polymerase-DNA complex can thermally fluctuate between pre- and post- translocated states.

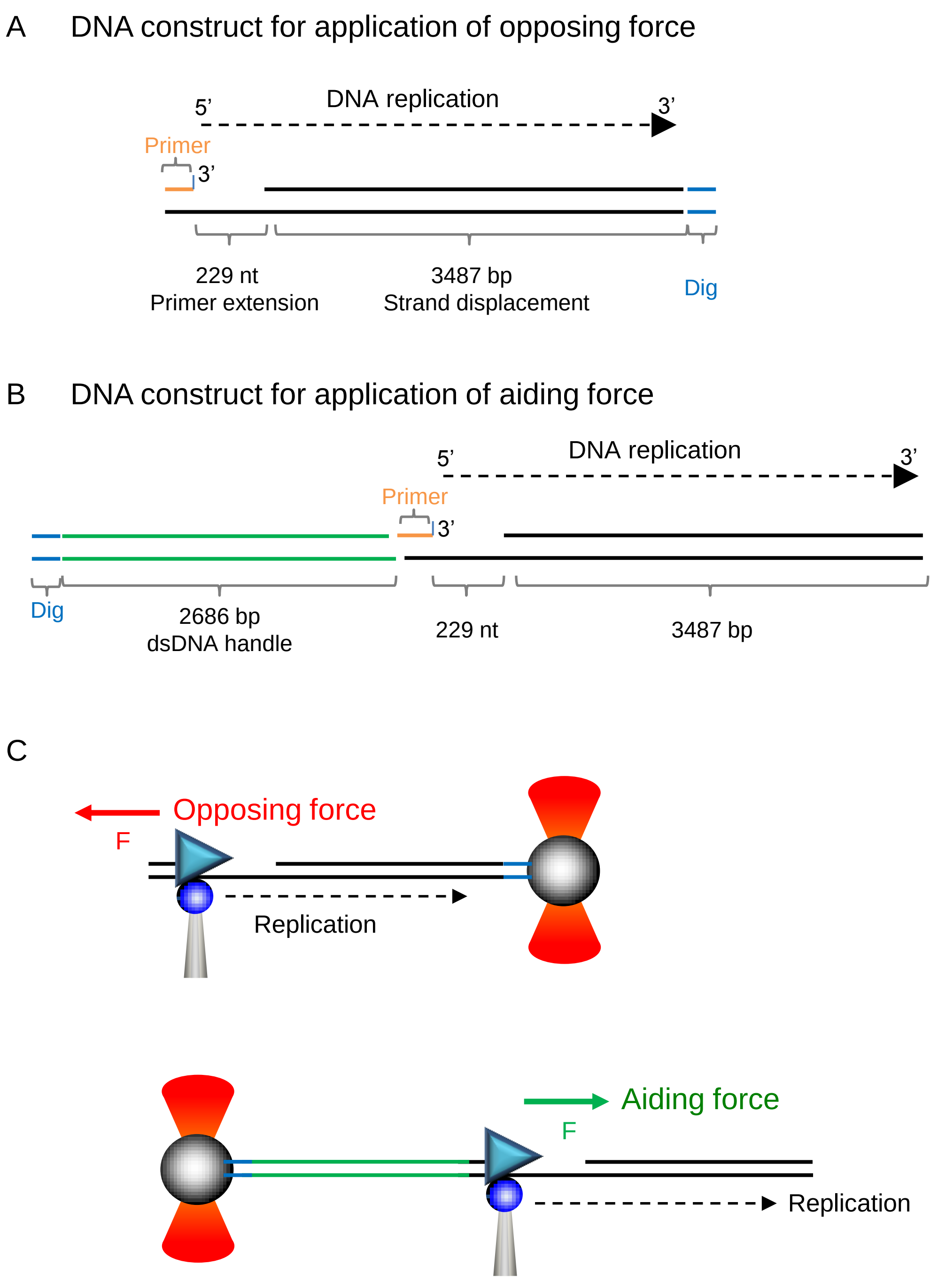

Supplementary Figure S1

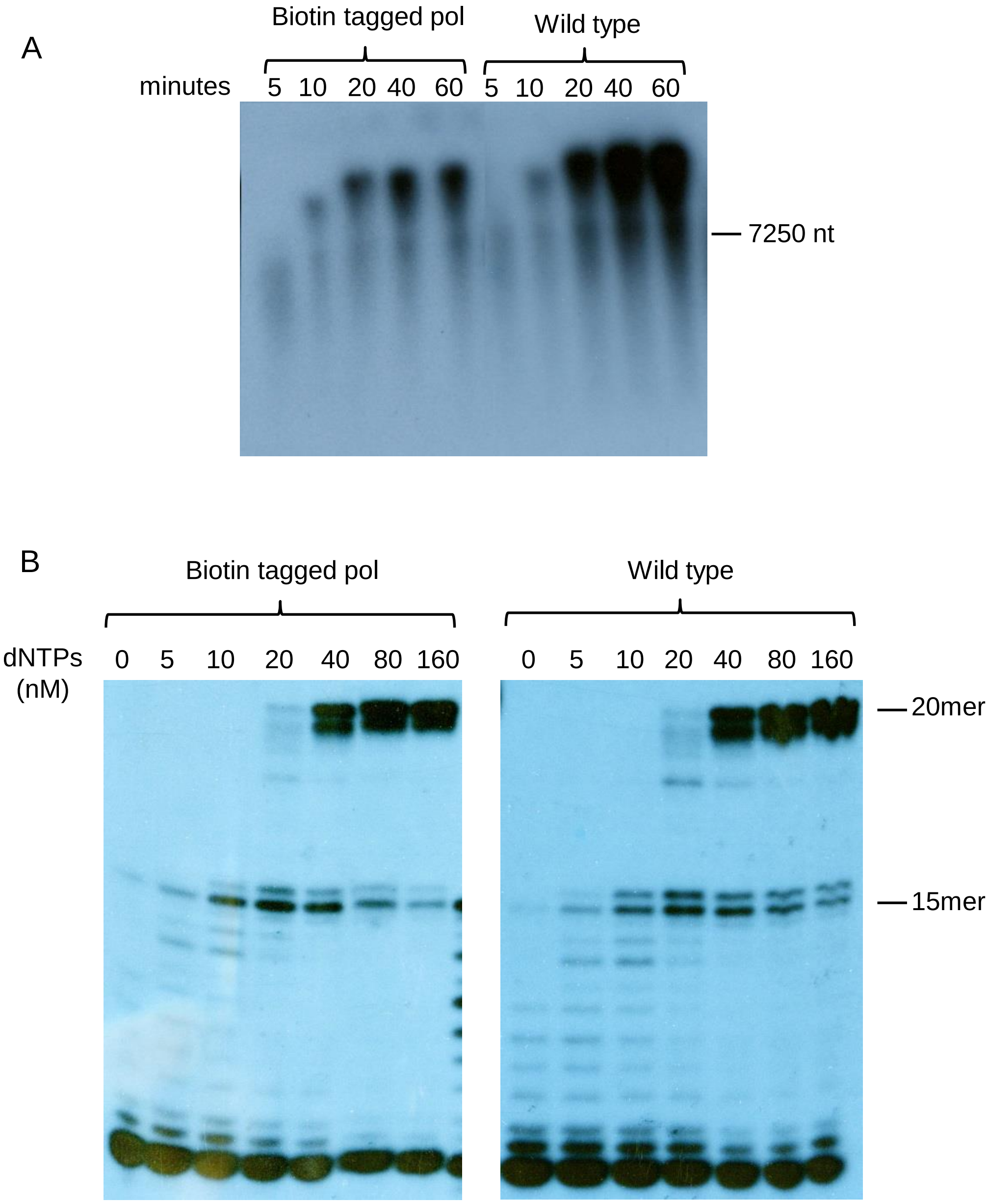

Supplementary Figure S2

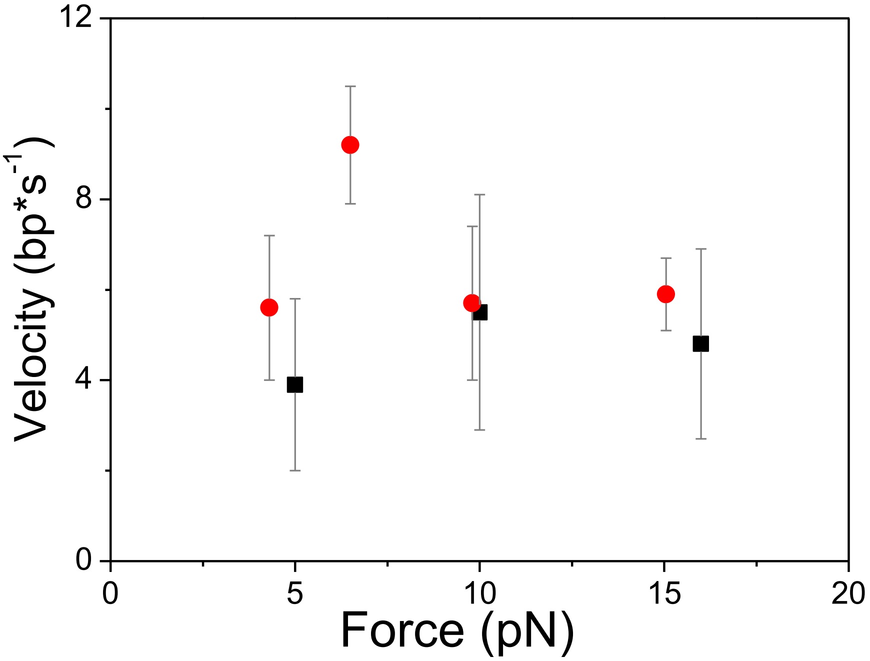

Supplementary Figure S3

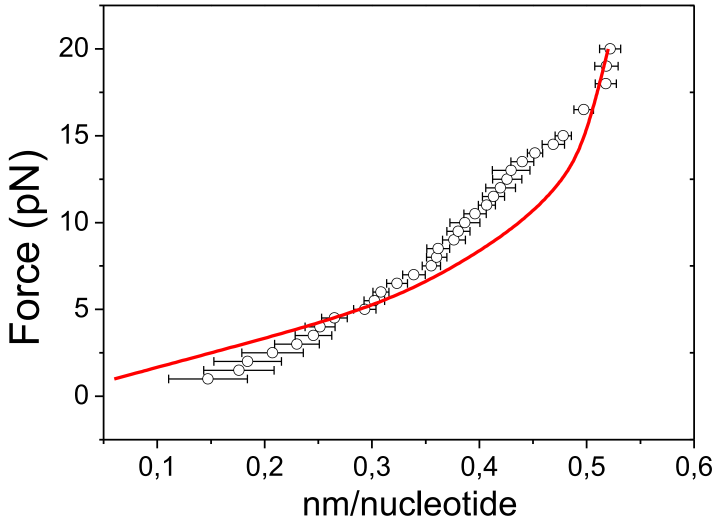

Supplementary Figure S4

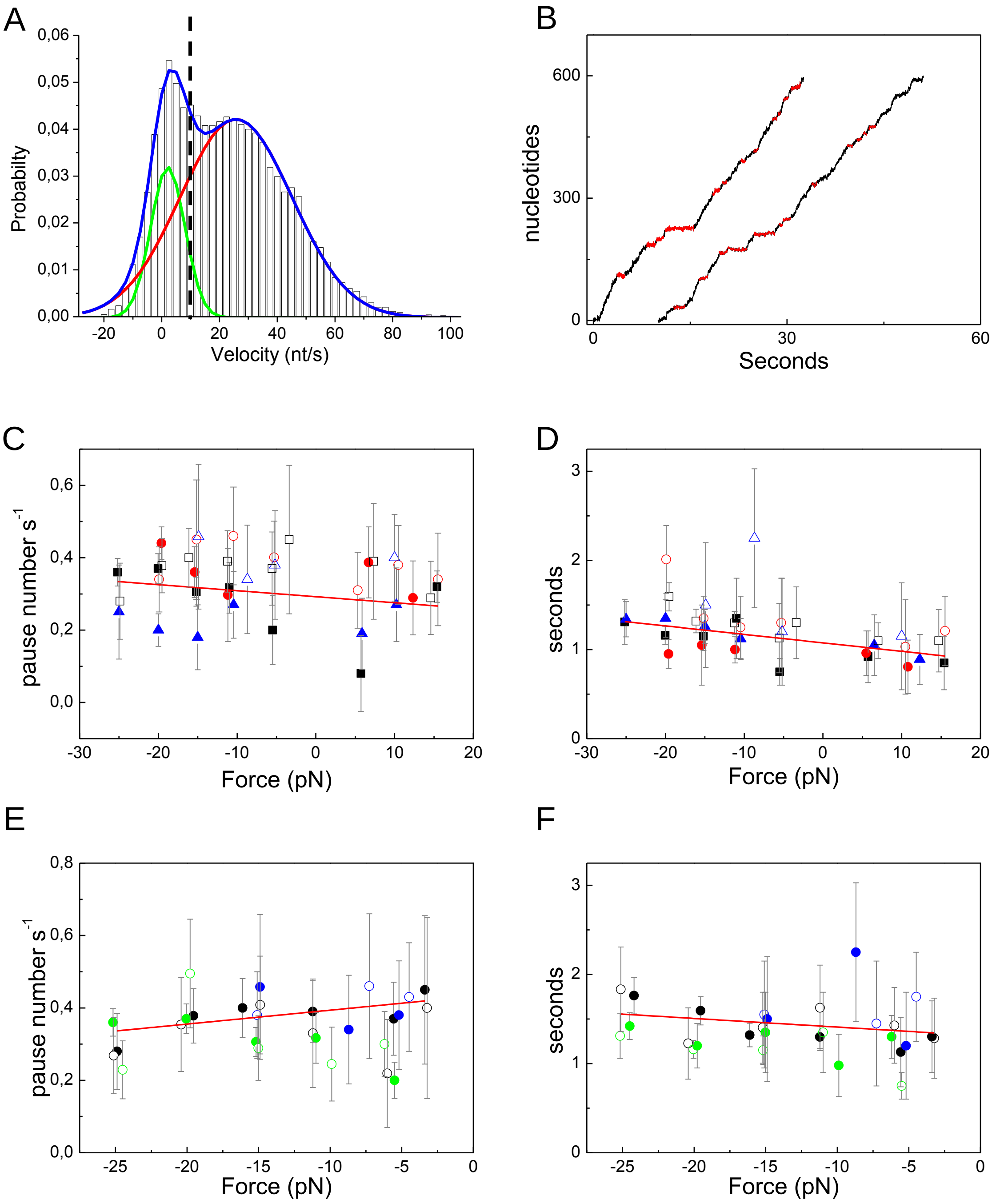

Supplementary Figure S5

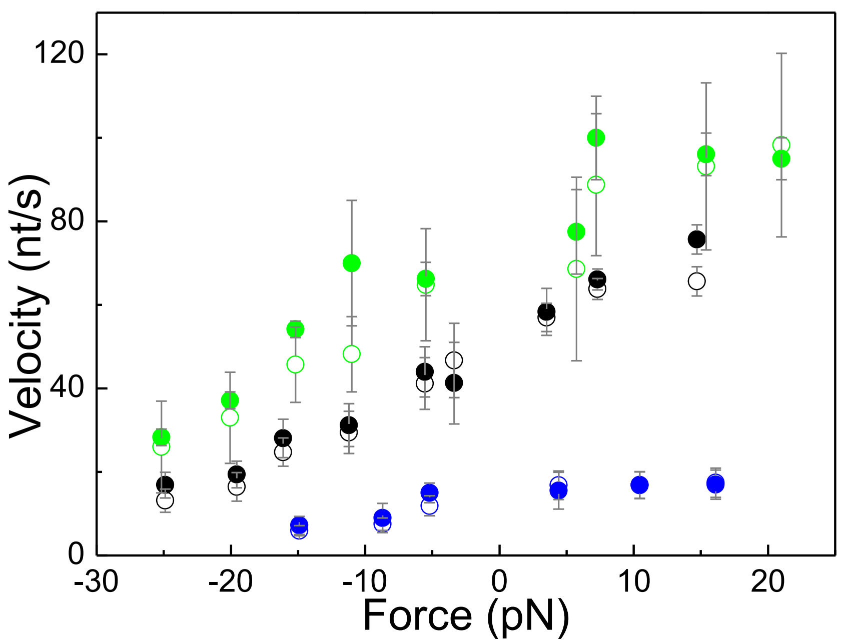

Supplementary Figure S6

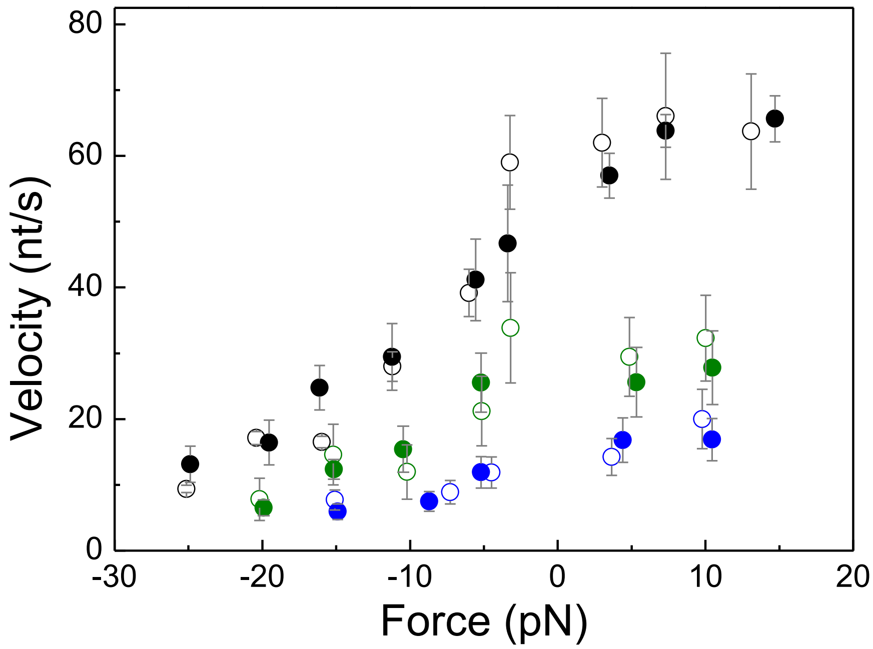

Supplementary Figure S7

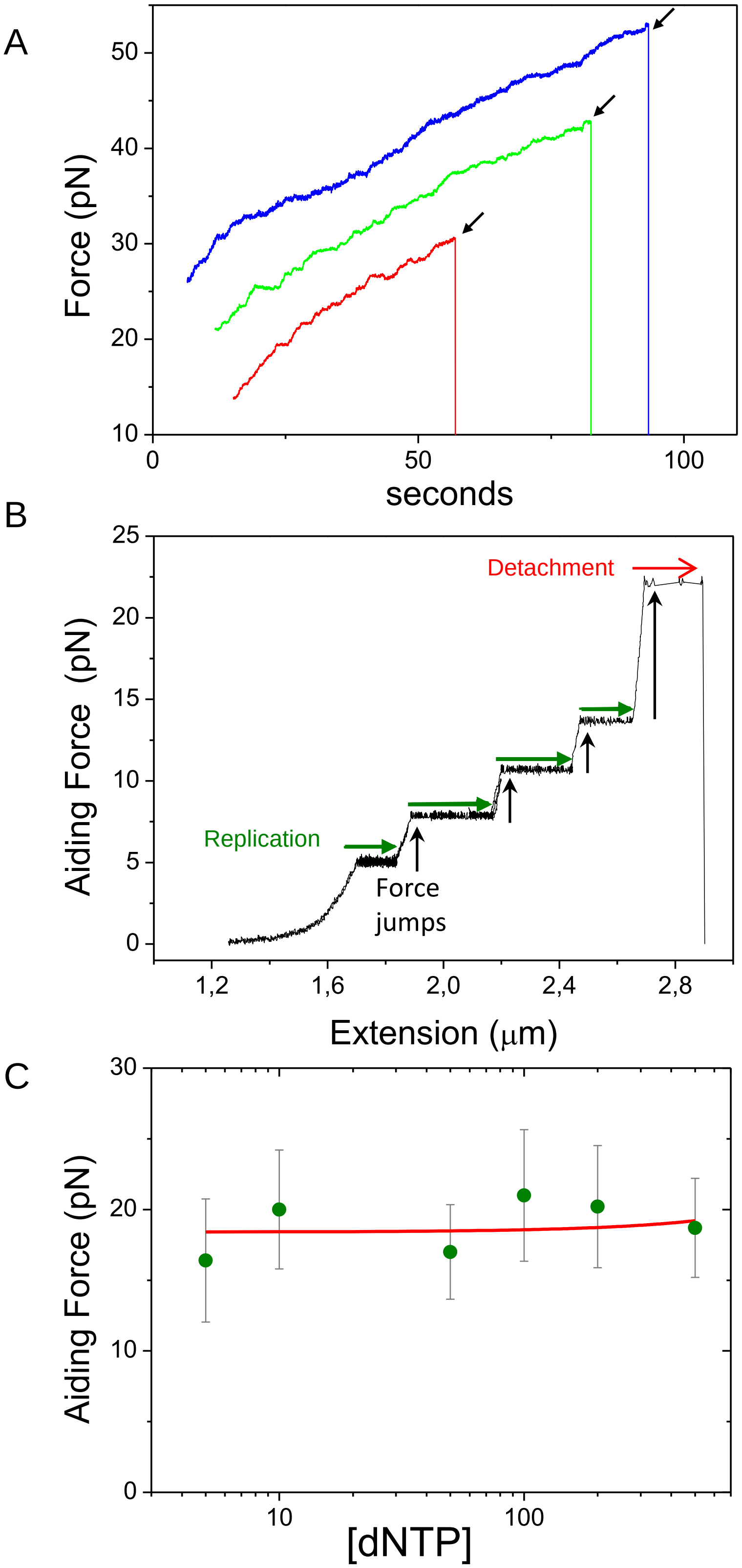

Supplementary Figure S8

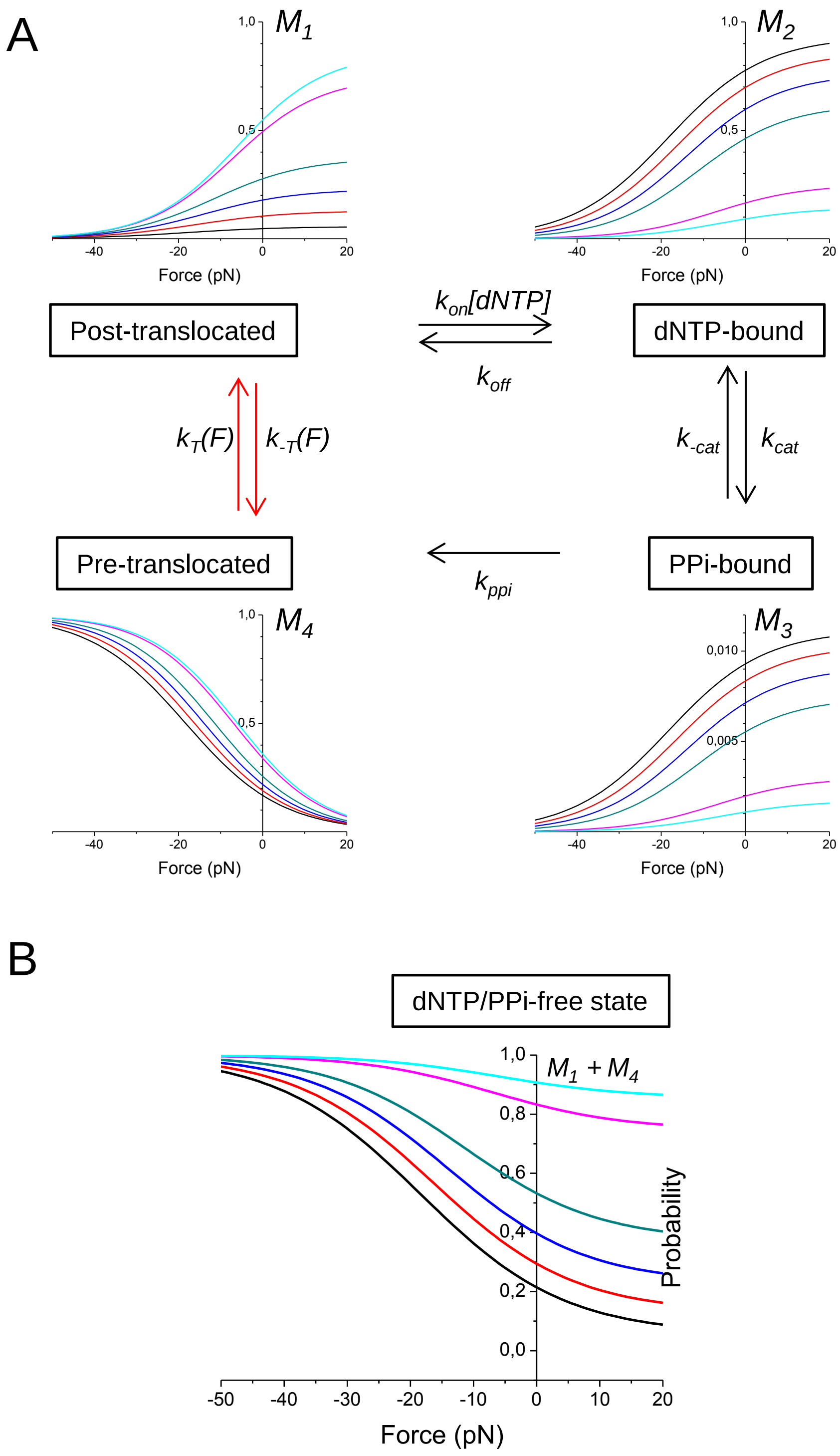

Supplementary Figure S9
